# Age-specific transmission dynamics under suppression control measures during SARS-CoV-2 Omicron BA.2 epidemic

**DOI:** 10.1186/s12889-023-15596-w

**Published:** 2023-04-22

**Authors:** Wenlong Zhu, Zexuan Wen, Yue Chen, Xiaohuan Gong, Bo Zheng, Xueyao Liang, Ao Xu, Ye Yao, Weibing Wang

**Affiliations:** 1 Shanghai Institute of Infectious Disease and Biosecurity, School of Public Health, Fudan University, 138 Yi Xue Yuan Road, Shanghai, 200032 China; 2 School of Epidemiology and Public Health, University of Ottawa, Ottawa, K1G5Z3 Canada; 3 Institute of Infectious Diseases, Shanghai Municipal Center of Disease Control and Prevention, Shanghai, 200336 China; 4 Key Laboratory of Public Health Safety of Ministry of Education, Fudan University, 138 Yi Xue Yuan Road, Shanghai, 200032 China

**Keywords:** COVID-19, Omicron variant, SEIR model, Transmission distance, Susceptibility and infectivity

## Abstract

**Background:**

From March to June 2022, an Omicron BA.2 epidemic occurred in Shanghai. We aimed to better understand the transmission dynamics and identify age-specific transmission characteristics for the epidemic.

**Methods:**

Data on COVID-19 cases were collected from the Shanghai Municipal Health Commission during the period from 20th February to 1st June. The effective reproductive number (R_t_) and transmission distance between cases were calculated. An age-structured SEIR model with social contact patterns was developed to reconstruct the transmission dynamics and evaluate age-specific transmission characteristics. Least square method was used to calibrate the model. Basic reproduction number (R_0_) was estimated with next generation matrix.

**Results:**

R_0_ of Omicron variant was 7.9 (95% CI: 7.4 to 8.4). With strict interventions, R_t_ had dropped quickly from 3.6 (95% CI: 2.7 to 4.7) on 4th March to below 1 on 18th April. The mean transmission distance of the Omicron epidemic in Shanghai was 13.4 km (95% CI: 11.1 to 15.8 km), which was threefold longer compared with that of epidemic caused by the wild-type virus in Wuhan, China. The model estimated that there would have been a total 870,845 (95% CI: 815,400 to 926,289) cases for the epidemic from 20th February to 15th June, and 27.7% (95% CI: 24.4% to 30.9%) cases would have been unascertained. People aged 50–59 years had the highest transmission risk 0.216 (95% CI: 0.210 to 0.222), and the highest secondary attack rate (47.62%, 95% CI: 38.71% to 56.53%).

**Conclusions:**

The Omicron variant spread more quickly and widely than other variants and resulted in about one third cases unascertained for the recent outbreak in Shanghai. Prioritizing isolation and screening of people aged 40–59 might suppress the epidemic more effectively. Routine surveillance among people aged 40–59 years could also provide insight into the stage of the epidemic and the timely detection of new variants.

**Trial registration:**

We did not involve clinical trial.

**Supplementary Information:**

The online version contains supplementary material available at 10.1186/s12889-023-15596-w.

## Background

Since severe acute respiratory syndrome coronavirus 2 (SARS-CoV-2) Omicron variant was designated as a variant of concern (VOC) by the World Health Organization (WHO) on 26th November 2021, it has been rapidly spreading and has become the dominant variant circulating globally [1]. Since the first Omicron case in Shanghai was reported on 20th February, the outbreak has resulted in more than 600,000 people being infected as of 1st June. Phylogenetic features of SARS-CoV-2 viral genomes from 129 patients, and inferring their relationship with those available on the GISAID database, indicated that the new viral genomes in Shanghai were clustered into the SARS-CoV-2 BA.2 sub-lineage [2]. To suppress the transmission based on the “Zero-COVID” policy, non-pharmaceutical interventions (NPIs) are implemented including physical distancing, mandatory use of face masks, mass nucleic acid amplification testing (NAAT)/rapid antigen testing (RAT) and quarantine/isolation. Even with the massive NAAT/RAT screening, there might have been unascertained/undetected cases, which might have influenced the transmission dynamics.

The spatial characteristics of Coronavirus disease 2019 (COVID-19) are of great significance for assessing transmission characteristics of the disease and for guiding implementation of interventions [3, 4]. Infectious individuals are often clustered in space during the initial phase of sustained transmission, which provides an opportunity to make effective use of limited resources for intervention [5]. Estimation of the transmission distance between cases contribute to targeting resources both for control and enhanced surveillance [6].

Age dependent susceptibility to SARS-CoV-2 infection as well as the infectiousness upon infection have a significant impact on implementation of public health interventions. Most studies suggested that the susceptibility of children was lower than that of adults and the elderly [7–10] or no significant difference in infectivity among different age groups [7, 8], while other studies found an increased transmissibility in adults or elderly people [11, 12]. The vaccination coverage varied with age, which might drive the transition of COVID-19 burden [13]. Assessing age-specific susceptibility and transmission risk is conductive to understand transmission dynamics and improve the effectiveness of interventions through prioritizing different interventions in different age groups.

In the current study, we investigated the dynamics of transmission during the Omicron epidemic in Shanghai between March and June 2022, estimated transmission distance between cases and developed an age-structured SEIR (Susceptible, Exposed, Infectious and Recovered) model to reconstruct the transmission dynamics for the Omicron epidemic in Shanghai, to estimate the susceptibility and transmissibility of different age groups and assess the age prioritization of implementation of interventions.

## Methods

### Data collection

COVID-19 infection data in Shanghai from 20th February to 1st June 2022 were from the daily reports released by the Shanghai Municipal Health Commission (https://wsjkw.sh.gov.cn/). Information on sex, age, residential address and clinical classification was only available for cases reported before 17th March. Parameters used in our model were form previous studies or estimated by the least square method (LSM), and were presented in Additional file 1: Table S1 [14–17].

### Age-structured SEIR model

**Fig. 1:**
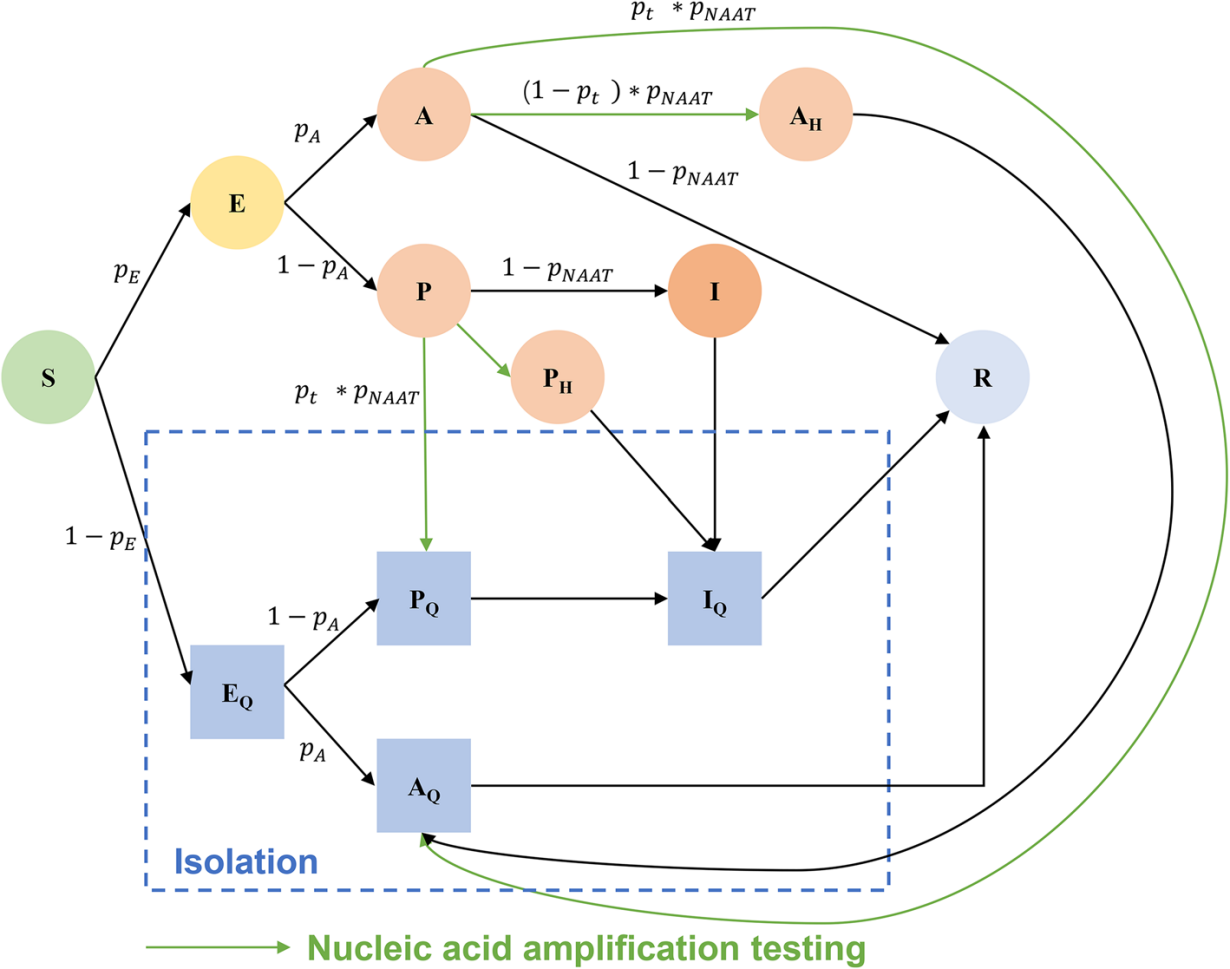
Flow patterns between different compartments in the model

An age-structured SEIR model considered age-structured differences in population mixing was developed. Based on the vaccination (Additional file 1: Role of vaccination [15, 18, 19]) and social contact characteristics (Additional file 1: Constructing social contact matrix [20, 21]), the population was divided into 10 age groups ($i$): 0–2, 3–11, 12–17, 18–29, 30–39, 40–49, 50–59, 60–69, 70–79 and 80 + . Susceptible (${S}^{i}$) infected by infectious cases (asymptomatic infection: ${A}^{i}$, presymptomatic infection: ${P}^{i}$, and symptomatic cases: ${I}^{i}$) would become exposed (${E}^{i}$ and ${E}_{Q}^{i}$). Exposed individuals were not infectious and not detected by NAAT/RAT. After the latent period ($\alpha$), a proportion (${p}_{A}$) of exposed individuals became asymptomatic infection (${A}^{i}$, ${A}_{Q}^{i}$), others became presymptomatic infection (${P}^{i}, {P}_{Q}^{i}$). After a certain period (${\alpha }_{2}$, incubation period – latent period), a presymptomatic infection become a symptomatic infection (${I}^{i}$, ${I}_{Q}^{i}$). All cases became recovered individuals (${R}^{i}$) if they were no longer contagious. The superscript $i$ refers to different age groups ($i=1, 2, \dots \dots , 10$), subscript $Q$ indicated that the individuals were isolated/treated and could not infect susceptible people. Asymptomatic (${A}^{i}$) and presymptomatic (${P}^{i}$) infection would be quarantined/isolated with positive NAAT results. In our model, we assumed that from 28th March to 12th April, a proportion ($1-{p}_{t}$) of asymptomatic and presymptomatic cases who were detected through NAAT would be self-isolation at home (${A}_{H}^{i}$, ${P}_{H}^{i}$) due to the limitation of centralized isolation resources (Additional file 1: Figure S1). ${A}_{H}^{i}$ and ${P}_{H}^{i}$ would only infect their family members (${CM}_{H}$). The model could be described in Fig. 1 and the equations in Additional file 1: Model calibration. All parameters of our model were presented in the Additional file 1: Table S1 [14–17].

### Statistical analysis

#### Estimating effective reproduction number

The effective reproductive number (R_t_) was calculated using the method developed by Cori er al [22] in R software version 4.1.2. The daily number of reported infections and the serial interval (mean: 3.5 days, standard deviation (SD): 2.4 days [17]) for the Omicron variant from a previous study [17] were used to estimate R_t_ and its 95% confidence interval (95% CI) on each day via a 7-day moving average. For sensitivity analysis, a 4-day moving average was used to estimate the R_t_.

#### Estimating transmission distance

Based on the location data of 1482 cases reported before 17th March and the generation time distribution (Gamma distribution with mean: 3.3 days and SD: 3.5 days [23]), we used the method developed by Salje et al. [6] to estimate the transmission distance between cases. We assumed the transmission distance distribution to follow a typical power law distribution, where the key parameter, λ, could be calculated by the mean transmission distance. To compare the transmission distance among different variants, cases in the initial period of Wuhan epidemic (8th December 2019 to 14th January 2020) were used to estimate the transmission distances. During the initial period of the epidemic in these two cities, few strict NPIs were implemented.

#### Calibrating the model and estimating the proportion of unascertained cases

With the number of daily reported cases, the least square method (LSM) was used to calibrate the model and to estimate the age-specific transmission rate (${\beta }^{i}$, Additional file 1: Model calibration). Transmission rate (${\beta }^{i}$) in this study is defined as probability that one contact between a case and a susceptible person will result in an effective infection. The next generation matrix (NGM) was used to estimate the basic reproduction number (R_0_, Additional file 1: Next generation matrix [24–26]).

In our model, unascertained cases refer to asymptomatic cases that are not traced and not detected by NAAT/RAT before they are not contagious. Based on the calibrated model, the cumulative number of unascertained cases (${Cuf}^{i}$) and all cases (${C}^{i}$, ascertained and unascertained) in each age group were calculated according to the following equations. The proportion of unascertained cases was calculated by the sum of $\frac{{dCuf}^{i}}{dt}$ divided by the sum of $\frac{{dC}^{i}}{dt}$.

$$\begin{array}{c}\frac{{dCuf}^i}{dt}=\frac{\beta^i\ast S^i\ast p_{nv}^i\ast\left[p_E\ast CM\ast\left({\varepsilon A}^i+{\varepsilon P}^i+I^i\right)+{CM}_H\ast\left(\varepsilon A_H^i+{\varepsilon P}_H^i\right)\right]}{Ni}\\-\frac{\mathrm pt\ast p_{NAAT}\ast\left(A^i+P^i\right)}{\alpha_3}-\frac{\left(A_H^i+P_H^i\right)}{\alpha_H}-\frac{I^i}{\alpha_4}\\\frac{{dC}^i}{dt}=\frac{\beta^i\ast S^i\ast p_{nv}^i\ast\left[p_E\ast CM\ast\left({\varepsilon A}^i+{\varepsilon P}^i+I^i\right)+{CM}_H\ast\left(\varepsilon A_H^i+{\varepsilon P}_H^i\right)\right]}{N^i}\end{array}$$

With the ${\beta }^{i}$ and SAR, we inferred the susceptibility and infectivity and tried to prioritize different age groups for centralized isolation and NAAT/RAT (Additional file 1: Inferring the susceptibility and infectivity).

To assess the robustness of our results, sensitivity analyses were carried out on the major parameters in the model (Additional file 1: Sensitivity analysis). Different values of these parameters were used to recalibrate the model to assess their effects on age-specific transmission rates (${\beta }^{i}$), proportion of cases found by NAAT/RAT, SARs, cumulative incidences, R_0_ and proportion of unascertained cases (Additional file 1: Table S2 and Figure S2). Additionally, we accessed the impacts of proportion of untraced cases (${p}_{E}$), proportion of asymptomatic cases (${p}_{A}$) and proportion of cases found by NAAT/RAT (${p}_{NAAT}$) on the proportion of unascertained cases.

Estimation of the transmission distance was performed in MATLAB 2021a. Development and calibration of the model, estimation of R_0_, the proportion of unascertained cases and secondary attack rate, sensitivity analysis and visualization of results were performed in R software version 4.1.2.

## Results

### Spatio-temporal diffusion characteristics

**Fig. 2:**
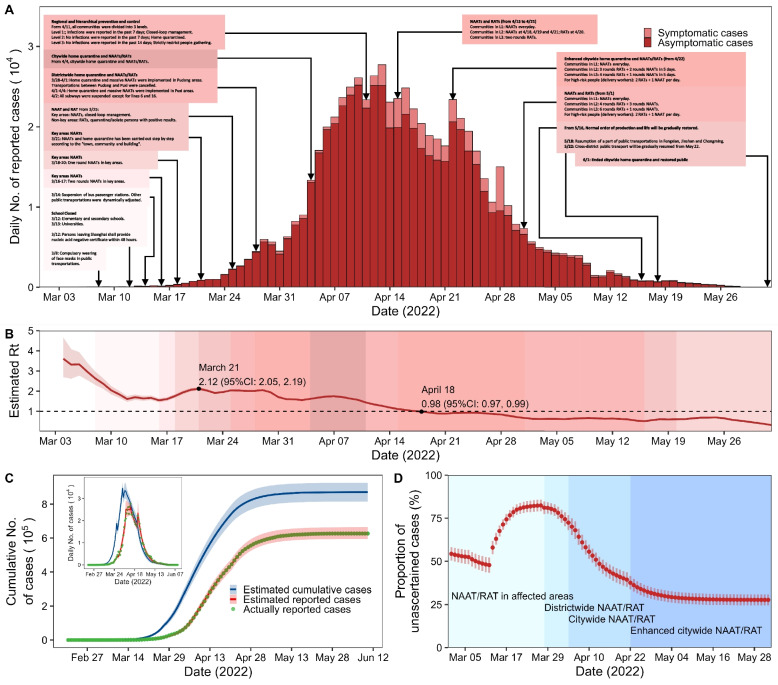
Epidemic curve of COVID-19 cases (**A**, **C**), effective reproductive number (R_t_, **B**) and time-varying proportion of unascertained cases (**D**) in the epidemic of Shanghai. **A** shows the epidemic curve of reported COVID-19 cases, key events, and public health interventions. **B** shows the effective reproductive number calculated from daily reported cases with 7-day moving average. **C** shows the epidemic curve of cumulative predicted cases and reported cases estimated by the model. **D** is the daily proportion of unascertained cases

From 20th February to 1st June, there were 626,825 reported COVID-19 cases in Shanghai, and among whom, 94.3% (591,358/626,825) were asymptomatic. The first home quarantine directive was implemented in six districts (Chongming, Fengxian, Jinshan, Minhang, Pudong and Songjiang) since 28th March, and a citywide home quarantine with mass screening of NAAT/RAT was put in place on 4 April. The number of daily reported cases peaked on 13 April (Fig. 2A). R_t_ decreased from 3.6 (95% CI: 2.7 to 4.7) on 4th March to 1.5 (95% CI: 1.4 to 1.6) on 16th March. R_t_ increased afterwards due to mass screening of NAAT/RAT and stabilized at 1.7 to 2.1 during the period from 17th March to 7th April. Since 7th April, the R_t_ gradually decreased and fell below 1 on 18th April (R_t_ = 0.98, 95% CI:0.97 to 0.99) (Fig. 2B). Based on our model, R_0_ of the Omicron epidemic in Shanghai was 7.9 (95% CI: 7.4 to 8.4). If COVID-19 vaccines were not available, R_0_ would increase to 9.6 (95% CI: 9.0 to 10.2).

Figure 2C shows the epidemic curves of cumulative predicted cases and reported cases for the epidemic. The model predicted the epidemic to end in mid-June with a total of 870,845 (95% CI: 815,400 to 926,289) cases, but among them, 241,104 (95% CI: 201,373 to 280,835) cases might be missed. We estimated that there had been 99,687 (95% CI: 85,020 to 114,355) cases on 28th March and 81.1% (95% CI: 77.4% to 84.8%) were unascertained (Fig. 2D).

**Fig. 3:**
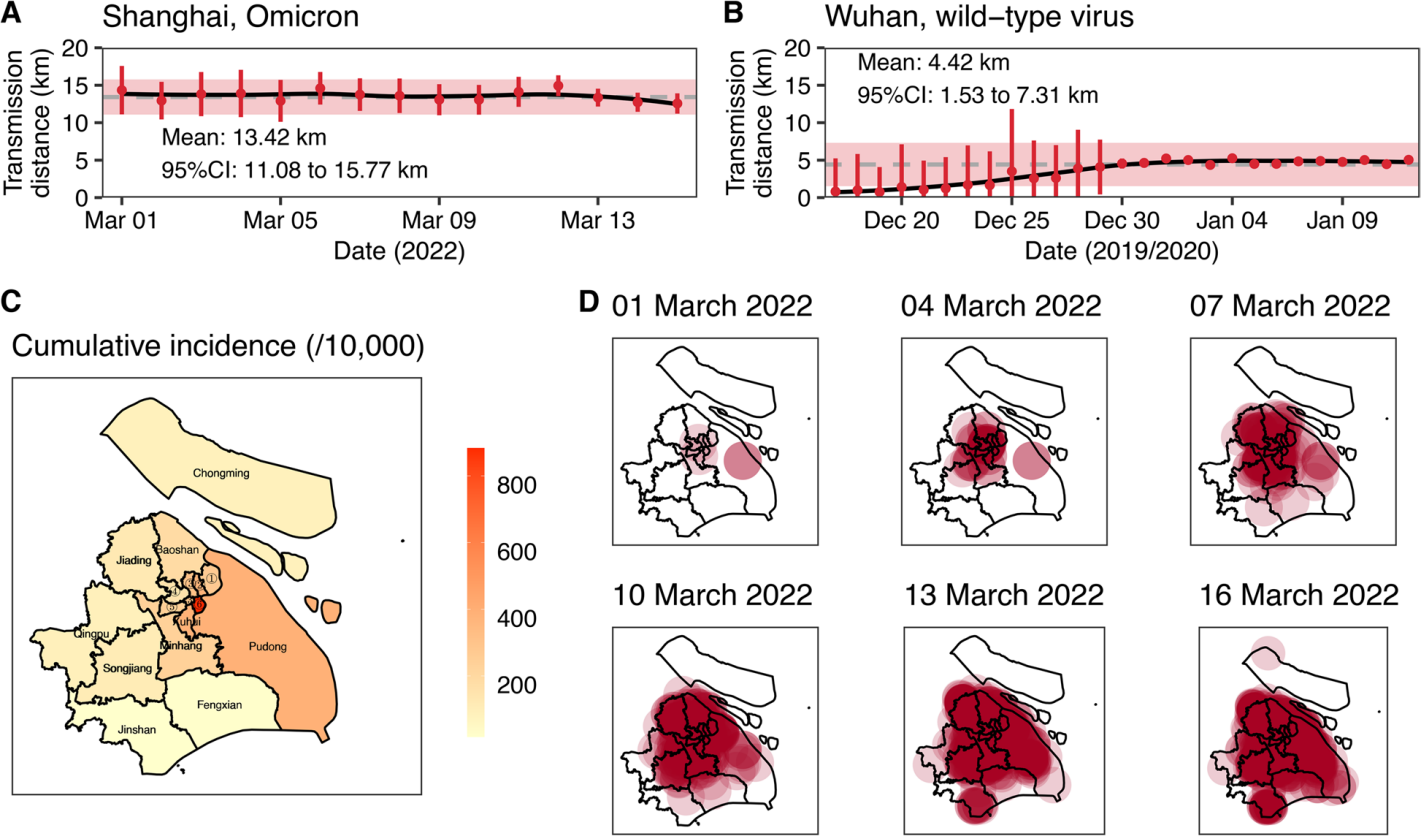
Cumulative incidence (**C**), time varying transmission distance (**A**, **B**) and the size of areas with infection risks (**D**). **A** and **B** is the time varying transmission distance of Shanghai and Wuhan respectively. Dashed grey line and the red shadow in **A** and **B** is the mean transmission distance and 95% confidence interval (95% CI) of mean transmission distance. Solid black line in **A** and **B** is smoothed with locally weighted regression. Central areas in **C** were: ① Yangpu, ② Hongkou, ③ Jing’an, ④ Putuo, ⑤ Changning, ⑥ Huangpu. **D** show the areas at potential risk of infection as of 1, 4, 7, 10, 13 and 16 March 2022 with 13.4 km transmission distance (the radius of each circle)

Pudong had the most reported infections (35.5%), followed by Minhang (10.1%), and Jinshan had the least reported infections (0.2%, Additional file 1: Figure S3). There were substantially geographic differences in the cumulative incidence (Fig. 3C). In the early stage of the epidemic, the mean transmission distance between cases was estimated to be 13.4 km (95% CI: 11.1 to 15.8) (Fig. 3A), which was threefold longer compared with the wild-type virus epidemic in Wuhan (4.4 km; 95% CI: 1.5 to 7.3; Fig. 3B). The transmission distance for the Shanghai epidemic varied from 12.5 km (95% CI: 11.2 to 13.9) on 15th March to 14.9 km (95% CI: 13.5 to 16.3) on 12th March. The transmission distance fluctuated around 13.4 km before 12th March, while decreased since school closure and suspension of public transportation started on 12th March. Based on the location of cases, Fig. 3D and Additional file 2: Movie S1 show that as of 13th March, the virus had spread widely across the city. Compared to the Wuhan epidemic for the date with the same cumulative number of cases, the size of epidemic areas with high risk levels was larger in Shanghai even with more strict intervention measures (Additional file 1: Figure S4).

### Change of contact patterns and age-specific transmission characteristics

**Fig. 4:**
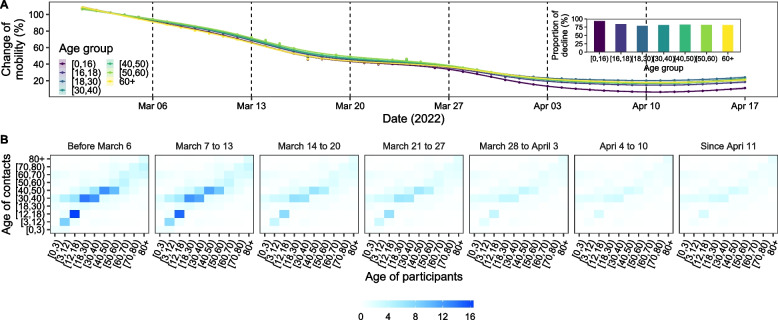
Mobility trends and estimated contract matrices in Shanghai. **A** shows the change of mobility trends for individuals aged 0–15, 16–17, 18–29, 30–39, 40–49, 50–59 and 60 + , relative to the baseline period 20–28 February, 2022. **B** shows the estimated social contact matrices for Shanghai population mixing during different periods

Population mobility had decreased gradually since the start of the epidemic (Fig. 4A). During the initial phase of the epidemic, it declined most markedly among individuals aged 0–16 and 60 + years and to the half of the original level after school closure (12th March). Since the implementation of citywide home quarantine, mobility declined to the minimum. Comparing to the mobility before the epidemic, children and adolescents had the greatest decline. Figure 4B shows that the frequency of human contacts declined for all the age group since the start of the outbreak. The decline was pronounced after 28th March, when home quarantine was implemented. Contact matrices at different settings over the seven periods were presented in Additional file 1: Figure S5.

**Fig. 5:**
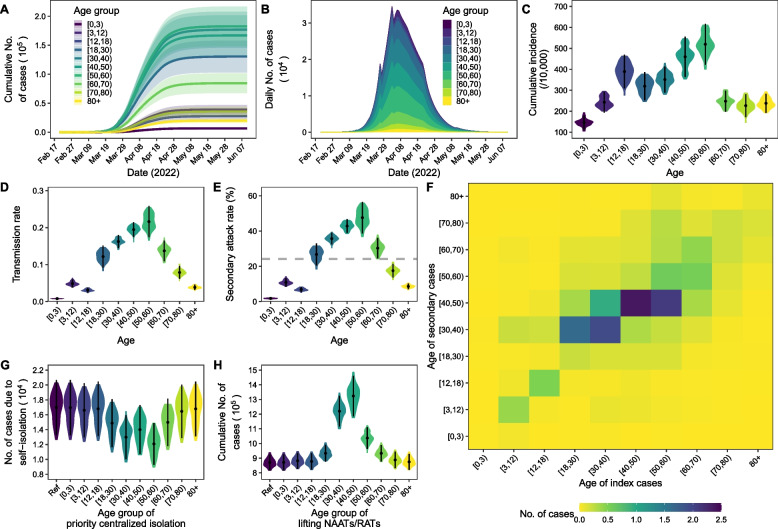
Epidemic curves (**A, B**), cumulative incidence (**C**) and distribution of transmission rates (**D**) in different ages. **E** shows the secondary attack rates (SARs) in different ages calculated based on the model estimated infections. Grey dashed line in E was the overall SAR (24.17%, 95% CI: 23.09% to 25.25%). **F** shows the age distribution of daily secondary cases infected by index cases in different ages. **G** is the number of secondary cases infected by index cases that are self-isolated at home. Ref in **G** is the scenario that all age groups of cases were self-isolated at home; other scenarios prioritize centralized isolation of cases in different age groups. **H** shows the distribution of the cumulative number of cases if the NAAT/RAT is lifted in each age group. Ref in **H** is the scenario that NAAT/RAT are implemented in all age groups

Among the 870,845 cases estimated for the epidemic, 60.5% (95% CI: 56.4% to 64.5%) were aged 30–59 years, 6.4% (95% CI: 5.3% to 7.4%) were aged 70 years or older, and 0.8% (95% CI: 0.6% to 1.0%) were children under 3 years of age (Fig. 5A, B). Compared to the wild-type virus epidemic of Wuhan, cases in the Omicron epidemic of Shanghai were younger (Additional file 1: Figure S6 [27, 28]). Population aged 50–59 years had the highest cumulative incidence (519.5/10,000, 95% CI: 422.1/10,00 to 616.9/10,000), followed by the 40–49-year age group (459.7/10,000, 95% CI: 364.0/10,000 to 555.4/10,000) and the 12–17-year age group (389.1/10,000, 95% CI: 309.0/10,000 to 469.4/10,000; Fig. 5C). The cumulative incidence was relatively low in children under 3 years of age (145.8/10,000, 95% CI: 111.1/10,000 to 180.6/10,000) and elderly people aged 70–79 years (226.7/10,000, 95% CI: 172.2/10,000 to 281.1/10,000). The transmission rate varied with age (Fig. 5D). Cases under the age of 3 years had the lowest transmission rate (0.008, 95% CI: 0.007 to 0.009). The transmission rate increased with age and peaked at the age of 50–59 years 0.216 (95% CI: 0.210 to 0.222) and then declined after 60 years. Figure 5E presents the model estimated SAR. The SAR was the lowest in children (0 to 2 years: 1.80%, 95% CI: 1.39% to 2.20%) and the highest in adults aged 50–59 years (47.62%, 95% CI: 38.71% to 56.53%). Cases of different ages were more likely to infect their peer groups (Fig. 5F). Cases of children and adolescents were also more likely to infect their parents or other family members. Cases aged 50 to 59 resulted in the most secondary cases (3.8 cases/day; 95% CI: 3.0 to 4.5 cases/day) followed by 40–49 years (3.5 cases/day; 95% CI: 3.1 to 3.9 cases/day) and 30–39 years (3.4 cases/day; 95% CI: 3.1 to 3.8 cases/day).

According to the susceptibility and infectivity of different ages, we analyzed the priority of centralized isolation and implementation of NAAT/RAT for different age groups. Prioritizing the centralized isolation of cases aged 50 to 59 years could reduce the largest number (28.8%, 95% CI: 23.3% to 34.3%) of secondary cases, followed by 30 to39 years (23.5%, 95% CI: 19.0% to 28.0%). Prioritizing the centralized isolation of children (0.05%, 95% CI: 0.04% to 0.06%) or elderly cases aged above 80 (1.0%, 95% CI: 0.8% to 1.3%) had less impact on reducing the number of secondary cases caused by self-isolation at home (Fig. 5G). Lifting mass NAAT/RAT for those aged 30 to 49 could result in more cases (30–39: 1,219,758, 95% CI: 1,115,473 to 1,324,042; 40–49: 1,323,445 95% CI: 1,185,313 to 1,461,577), while lifting mass NAAT/RAT for those aged 0–17 and 70 years above only had a slight influence on the cumulative number of cases (Fig. 5H).

### Sensitivity analysis

To assess the impact of different moving average on the estimation of R_t_, a 4-day moving average was used to estimate the R_t_ (Additional file 1: Figure S7). R_t_ estimated from 4-day moving average were similar to that estimated from 7-day moving average. R_t_ estimated from 4-day moving average fell below 1 on 18th March.

**Fig. 6:**
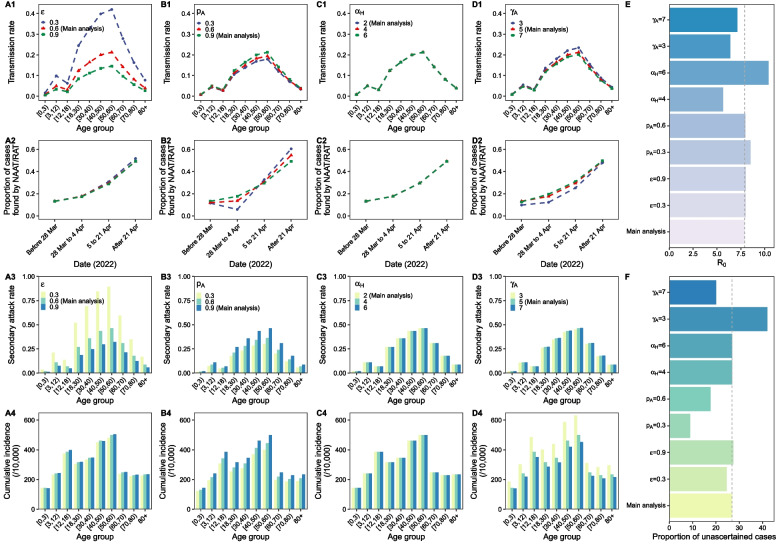
Impacts of the relative transmissibility rate of asymptomatic cases to symptomatic cases ($\varepsilon$, A1-A4), proportion of asymptomatic cases (${p}_{A}$, B1-B4), period of self-isolation at home (${\alpha }_{H}$, C1-C4) and recovery period of asymptomatic cases (${\gamma }_{A}$, D1-D4) on the transmission rates ($\beta$, first row), proportion of cases found by NAAT/RAT (${p}_{NAAT}$, second row), secondary attack rates ($SAR$, third row), cumulative incidences (fourth row), basic reproduction number (${R}_{0}$, **E**) and proportion of unascertained cases (**F**). Grey dashed line in E is the R_0_ reported in main analysis (7.9). Grey dashed line in F is the proportion of unascertained cases estimated in main analysis (27.7%)

To explore the robustness of our model and results, the relative transmissibility rate of asymptomatic cases to symptomatic cases ($\varepsilon$), proportion of asymptomatic cases (${p}_{A}$), period of self-isolation at home (${\alpha }_{H}$) and recovery period of asymptomatic cases (${\gamma }_{A}$) were included in the sensitivity analysis (Fig. 6). The model was well calibrated with different values of these parameters (Additional file 1: Figure S8). Most of the outcomes were not sensitive to the changes of these parameters. Transmission rate (${\beta }^{i}$) and ${SAR}^{i}$ increased with the decreasing of $\varepsilon$, but were stable to the change of other three parameters. Proportion of cases found by NAAT/RAT was stable to the change of these parameters. Cumulative incidence decreased with the increasing of ${\gamma }_{A}$. Basic reproduction number (R_0_) was sensitive to ${\alpha }_{H}$ and ${\gamma }_{A}$. Proportion of unascertained cases decreased with the reducing of ${p}_{A}$ and with the increasing of ${\gamma }_{A}$. Additionally, we accessed the impacts of proportion of untraced cases (${p}_{E}$) and proportion of cases found by NAAT/RAT (${p}_{NAAT}$) on the proportion of unascertained cases by sensitivity analysis. Results suggested that the proportion of unascertained cases increased with the increasing of ${p}_{E}$, but declined with the increasing of ${p}_{NAAT}$ (Additional file 1: Figure S9).

## Discussion

Based on the epidemic of Shanghai, we estimated that the R_0_ of Omicron variant was 7.9, comparable to the results of previous studies [29]. At the beginning of the epidemic, R_t_ was estimated to be 3.6, consistent with the results of previous studies (2.43 to 5.11) [29–31]. For both R_0_ and R_t_, Omicron variant were 3- to fourfold higher than these of Delta variant [29–31]. Increasing basic/effective reproductive number of Omicron variant have led to a rebound of the epidemic in numerous countries. The Omicron variant is highly transmissible with a high growth advantage over other variants, which is mainly driven by immune evasion [1, 32, 33]. From the reported data, the proportion of asymptomatic cases in the epidemic of Shanghai reached 90%. People infected with the Omicron variant have milder symptoms and fewer hospitalizations than those with other variants [34, 35], which lead to high covertness of this variant. The cumulative number of cases in the Omicron epidemic of Shanghai was estimated to be 870,845, and only 72.2% of them would have been ascertained. We estimated that there were 85.1% cases that were unascertained, before the implementation of home quarantine directive on 28th March. A previous study of the Wuhan epidemic indicated that 86% of all the infections were unascertained before the 23rd January 2020 travel restrictions [36], where suspension of public transportation and NAAT/RAT were not implemented before travel restrictions in Wuhan [27]. Additionally, it took 46 days in Wuhan and 36 days in Shanghai from the first case reported (8th December 2019 in Wuhan [37] and 20th February 2022 in Shanghai) to reach the same proportion of unascertained cases. Due to the highly transmissible and covert nature of the Omicron variant, mass vaccination and appropriate measures are necessary to decrease transmission and reduce mortality.

The Omicron variant also appeared to be spreading more widely, reflected by the mean transmission distance. During the initial period of the epidemic, the mean transmission distance of the Omicron epidemic in Shanghai was threefold longer than that of the wild-type virus epidemic in Wuhan. The longer transmission distance may indicate a more rapid spatial transmission of Omicron variant than the wild-type virus. The transmission distance of the epidemic in Shanghai decreased slightly since school closure and suspension of public transportation started on 12th March, reflected the impacts of NPIs on controlling the transmission of virus. Before 7th March, the spread of the virus was relatively localized and had not yet affected the entire city. These results suggested that earlier implementing NPIs may contribute to curbing the spatial transmission of the virus and reduce infections. The spatial spread of the virus can be determined by the estimation of the transmission distance, which contributes to targeting resources both for control and enhanced surveillance [6].

We estimated 60.1% cases were aged 30–59 years, 5.4% were children under the age of 12 years, and 2.4% were aged 80 years or older. The age for the cases of Shanghai epidemic tended to be younger than those in the epidemic of Wuhan [27, 28] and other localized outbreaks[38]. Cases infected with Omicron variant were found to be younger than cases infected with other variants [35], partly due to COVID-19 vaccination [13]. The overall SAR of the epidemic was estimated to be 24.17% (95% CI: 23.09% to 25.25%), which was consistent with previous studies [39–42]. Compared to observational studies, our SARs of children, adolescents, adults aged 60 years and above are lower, while SARs of adults aged 18–60 years are higher. SARs from observational studies might be biased by the ability of close contact tracking and detection of infections. For children and elderly, the number of close contacts they report is likely to be underestimated, which may result high SARs. Besides, it is difficult to detect all infections even massive nucleic acid testing was implemented. The underestimated number of infections might lead to a low SAR. In our analysis, if the SARs was calculated using the number of detectable cases (reported cases), the overall SAR reduced to 17.49% (95% CI: 16.25% to 18.71%, Additional file 1: Figure S10).

There seems to be no consensus about infectivity of different ages. Some studies indicated that the differences of infectivity among different age groups were not significant [7, 8], while the other studies reported an increased transmissibility among adults and elderly people [11, 12]. Besides, previous studies suggested that the susceptibility increased with age [8, 11, 43]. In this study, both transmission rate and SAR increased with age and peaked for people aged 50–59 years. People aged 50–59 report higher daily contacts, even during the lockdown period [21], which might lead to higher risk of infectivity and susceptibility. Additionally, patients aged 50–64 yeas seemed to have the long viral shedding time [44, 45] and higher viral load [46], which may associate with higher transmission rate.

Age-specific transmission rate and SAR are crucial to guide policymakers in implementing targeted COVID-19 control measures. Although the transmission rate and SAR in children and adolescents were lower, the transmissibility of them to other household members is not negligible. Extending vaccination to children aged 3–11 years would protect them from infection and reduce the transmission of this age group [13]. Cases with self-isolation at home could still infect susceptible people, such as their family members or neighbors [7, 47]. Prioritizing the isolation of cases aged 30–59 years and implementation of NAAT among population aged 30–49 years might curb the virus transmission and reduce the number of cases more efficiently. Routine surveillance among people aged 40–59 years could also provide insight into the stage of the epidemic and the timely detection of new variants. Promoting vaccination, encouraging mask-wearing and physical distancing, particularly among people aged 30–49 years, are still effective measures to reduce COVID-19 transmission and mortality.

This study was limited by the absence of detail information of the cases. The age distribution of cases in the early stage of the epidemic was used in the model calibration, which might result in biased estimation, especially for the age-specific transmission rate. We used the social contact matrix that was produced during 2019, and people’s contact habits might be reshaped by the COVID-19 pandemic and some intervention measures. Many parameters used in the model are assumed to be constant respect to time and age, but our sensitivity analysis suggested that our model and results were stable to the changes of these parameters. Detail information of cases and time-varying/age-specific parameters could allow us to provide a better reconstruction of the Shanghai’s Omicron epidemic and reduce the biases of results. Besides, the transmission distances may be affected by people's activities and the development level of the city. To further explore the regularity of the COVID-19 spatial transmission, more cities and variables should be included in the future studies.

## Conclusions

The Omicron variant presented a higher transmissibility and spread more widely and quickly than other variants, which might have resulted in about one third positive cases undetected in this wave of epidemic in Shanghai. People aged 50–59 years had the highest transmission risk and secondary attack rate. Prioritizing centralized isolation and screening of people aged 40–59 years might have suppressed the epidemic more effectively. Routine surveillance among these age groups may also provide insight into the stage of the epidemic and the timely detection of new variants.

## Supplementary Information

**Additional file 1:** **Table S1.** Meanings and initial values of parameters in the age-structured SEIR model. **Table S2.** Outcomes, parameters and their values used in sensitivity analysis. **Figure S1.** Patterns of parameters (p_E, p_NAAT, and p_t) variation with date. A: Proportion of untraced exposed persons (p_E). B: Proportion of cases found by NAAT/RAT (p_NAAT). C: Proportion of concentrated isolation among positive NAAT cases (p_t). **Figure S2.** Values and regularity of p_E (A) and p_NAAT (B) used in sensitivity analysis. **Figure S3.** Cumulative number of cases in different districts of Shanghai. **Figure S4.** The potential areas of virus had been spread on different date of the epidemics in Shanghai (A) and Wuhan (B) with different mean transmission distance (namely the radius of each circle: 13.4 km in Shanghai and 4.4 km in Wuhan). The cumulative number of cases was same for each column. **Figure S5.** Estimated contract matrices at different settings over seven periods in Shanghai. **Figure S6.** Demographic comparison of population (A), cases (B) and standard incidence (C) for Shanghai and Wuhan. **Figure S7.** Effective reproductive number (Rt) in the epidemic of Shanghai. Rt was estimated from4-day and 7-day moving average respectively. **Figure S8.** Epidemic curve of cumulative predicted cases and reported cases estimate by the model with different values of parameters. **Figure S9.** The proportion of unascertained cases under different proportion of asymptomatic cases (A), proportion of untraced cases (B) and proportion of cases detected by NAAT/RAT (C). **Figure S10.** Comparation of estimated secondary attack rates (SAR) and estimated reported SAR (SARobs). Blue and red dashed line is overall SAR (24.1%) and overall SARobs (17.4%).

**Additional file 2.** .

## Data Availability

The aggregated data on the daily reported number of COVID-19 cases are available on Github: https://github.com/wwbgroup/Shanghai2022Covid. Location data of each case reported before 17 March and codes used during the current study are available from the corresponding author on reasonable request.

## Abbreviations

SARS-CoV-2: Severe acute respiratory syndrome coronavirus 2
VOC: Variant of concern
WHO: World health organization
NPI: Non-pharmaceutical intervention
NAAT: Nucleic acid amplification testing
RAT: Rapid antigen testing
COVID-19: Coronavirus disease 2019
SEIR: Susceptible exposed infectious recovered
R_t_: Effective reproductive number
95% CI: 95% confidence interval
LSM: Least square method
NGM: Next generation matrix
R_0_: Basic reproduction number
RSS: Residual sum of squares
SAR: Secondary attack rate
km: Kilometer

## References

[1] WHO. Weekly epidemiological update on COVID-19 - 4 May 2022 https://www.who.int/publications/m/item/weekly-epidemiological-update-on-covid-19---4-may-2022. Accessed 10 May 2022.

[2] Zhang X, Zhang W, Chen S. Shanghai’s life-saving efforts against the current omicron wave of the COVID-19 pandemic. Lancet. 2022;399(10340):2011–2. 35533708 10.1016/S0140-6736(22)00838-8 PMC9075855

[3] Zhou S, Zhou S, Zheng Z, Lu J. Optimizing spatial allocation of COVID-19 vaccine by agent-based spatiotemporal simulations. Geohealth. 2021;5(6):e2021GH000427. 34179672 10.1029/2021GH000427 PMC8207830

[4] Liu W, Wang D, Hua S, Xie C, Wang B, Qiu W, et al. Spatiotemporal analysis of COVID-19 outbreaks in Wuhan, China. Sci Rep. 2021;11(1):13648. 34211038 10.1038/s41598-021-93020-2 PMC8249501

[5] Riley S. Large-scale spatial-transmission models of infectious disease. Science. 2007;316(5829):1298–301. 17540894 10.1126/science.1134695

[6] Salje H, Cummings DAT, Lessler J. Estimating infectious disease transmission distances using the overall distribution of cases. Epidemics. 2016;17:10–8. 27744095 10.1016/j.epidem.2016.10.001 PMC5159225

[7] Hu S, Wang W, Wang Y, Litvinova M, Luo K, Ren L, et al. Infectivity, susceptibility, and risk factors associated with SARS-CoV-2 transmission under intensive contact tracing in Hunan, China. Nat Commun. 2021;12(1):1533. 33750783 10.1038/s41467-021-21710-6 PMC7943579

[8] Sun K, Wang W, Gao L, Wang Y, Luo K, Ren L, et al. Transmission heterogeneities, kinetics, and controllability of SARS-CoV-2. Science. 2021;371(6526):eabe2424. 33234698 10.1126/science.abe2424 PMC7857413

[9] Zhao Z, Chen Q, Wang Y, Chu M, Hu Q, Hannah MN, et al. Relative transmissibility of shigellosis among different age groups: A modeling study in Hubei Province, China. PLoS Negl Trop Dis. 2021;15(6):e0009501. 34111124 10.1371/journal.pntd.0009501 PMC8219151

[10] Viner RM, Mytton OT, Bonell C, Melendez-Torres GJ, Ward J, Hudson L, et al. Susceptibility to SARS-CoV-2 Infection Among Children and Adolescents Compared With Adults: A Systematic Review and Meta-analysis. JAMA Pediatr. 2021;175(2):143–56. 32975552 10.1001/jamapediatrics.2020.4573 PMC7519436

[11] Franco N, Coletti P, Willem L, Angeli L, Lajot A, Abrams S, et al. Inferring age-specific differences in susceptibility to and infectiousness upon SARS-CoV-2 infection based on Belgian social contact data. PLoS Comput Biol. 2022;18(3):e1009965. 35353810 10.1371/journal.pcbi.1009965 PMC9000131

[12] Zhao ZY, Zhu YZ, Xu JW, Hu SX, Hu QQ, Lei Z, et al. A five-compartment model of age-specific transmissibility of SARS-CoV-2. Infect Dis Poverty. 2020;9(1):117. 32843094 10.1186/s40249-020-00735-x PMC7447599

[13] Cai J, Yang J, Deng X, Peng C, Chen X, Wu Q, et al. Assessing the transition of COVID-19 burden towards the young population while vaccines are rolled out in China. Emerg Microbes Infect. 2022;11(1):1205–14. 35380100 10.1080/22221751.2022.2063073 PMC9045766

[14] Statistics Bureau of Shanghai Municipality. https://tjj.sh.gov.cn/. Accessed 1 Mar 2022.

[15] Shanghai Municipal Health Commission. https://wsjkw.sh.gov.cn/. Accessed 1 Feb 2023.

[16] Gao W, Lv J, Pang Y, Li LM. Role of asymptomatic and pre-symptomatic infections in covid-19 pandemic. BMJ. 2021;375:n2342. 34852994 10.1136/bmj.n2342 PMC8634419

[17] Backer JA, Eggink D, Andeweg SP, Veldhuijzen IK, van Maarseveen N, Vermaas K, et al. Shorter serial intervals in SARS-CoV-2 cases with Omicron BA.1 variant compared with Delta variant, the Netherlands, 13 to 26 December 2021. Euro Surveill. 2022;27(6):2200042. 35144721 10.2807/1560-7917.ES.2022.27.6.2200042 PMC8832521

[18] Huang Z, Xu S, Liu J, Wu L, Qiu J, Wang N, et al. Effectiveness of inactivated and Ad5-nCoV COVID-19 vaccines against SARS-CoV-2 Omicron BA. 2 variant infection, severe illness, and death. BMC Med. 2022;20(1):400. 36266697 10.1186/s12916-022-02606-8 PMC9583051

[19] Palacios R, Patino EG, de Oliveira PR, Conde M, Batista AP, Zeng G, et al. Double-blind, randomized, placebo-controlled phase III clinical trial to evaluate the efficacy and safety of treating healthcare professionals with the adsorbed COVID-19 (Inactivated) vaccine manufactured by sinovac - PROFISCOV: A structured summary of a study protocol for a randomised controlled trial. Trials. 2020;21(1):853. 33059771 10.1186/s13063-020-04775-4 PMC7558252

[20] Zhang J, Klepac P, Read JM, Rosello A, Wang X, Lai S, et al. Patterns of human social contact and contact with animals in Shanghai, China. Sci Rep. 2019;9(1):15141. 31641189 10.1038/s41598-019-51609-8 PMC6805924

[21] Zhang J, Litvinova M, Liang Y, Wang Y, Wang W, Zhao S, et al. Changes in contact patterns shape the dynamics of the COVID-19 outbreak in China. Science. 2020;368(6498):1481–6. 32350060 10.1126/science.abb8001 PMC7199529

[22] Cori A, Ferguson NM, Fraser C, Cauchemez S. A new framework and software to estimate time-varying reproduction numbers during epidemics. Am J Epidemiol. 2013;178(9):1505–12. 24043437 10.1093/aje/kwt133 PMC3816335

[23] Abbott S, Sherratt K, Gerstung M, Funk S. Estimation of the test to test distribution as a proxy for generation interval distribution for the Omicron variant in England. medRxiv. 2022;10:2022–01.

[24] Diekmann O, Heesterbeek JA, Roberts MG. The construction of next-generation matrices for compartmental epidemic models. J R Soc Interface. 2010;7(47):873–85. 19892718 10.1098/rsif.2009.0386 PMC2871801

[25] van den Driessche P, Watmough J. Reproduction numbers and sub-threshold endemic equilibria for compartmental models of disease transmission. Math Biosci. 2002;180:29–48. 12387915 10.1016/s0025-5564(02)00108-6

[26] Jaouimaa FZ, Dempsey D, Van Osch S, Kinsella S, Burke K, Wyse J, et al. An age-structured SEIR model for COVID-19 incidence in Dublin, Ireland with framework for evaluating health intervention cost. PLoS ONE. 2021;16(12):e0260632. 34874981 10.1371/journal.pone.0260632 PMC8651129

[27] Pan A, Liu L, Wang C, Guo H, Hao X, Wang Q, et al. Association of public health interventions with the epidemiology of the COVID-19 outbreak in Wuhan. China JAMA. 2020;323(19):1915–23. 32275295 10.1001/jama.2020.6130 PMC7149375

[28] Wu Z, McGoogan JM. Characteristics of and important lessons from the coronavirus disease 2019 (COVID-19) outbreak in China: summary of a report of 72314 Cases from the Chinese center for disease control and prevention. JAMA. 2020;323(13):1239–42. 32091533 10.1001/jama.2020.2648

[29] Liu Y, Rocklov J. The effective reproductive number of the Omicron variant of SARS-CoV-2 is several times relative to Delta. J Travel Med. 2022;29(3):037. 10.1093/jtm/taac037 PMC8992231 35262737

[30] Ito K, Piantham C, Nishiura H. Relative instantaneous reproduction number of Omicron SARS-CoV-2 variant with respect to the Delta variant in Denmark. J Med Virol. 2022;94(5):2265–8. 34967453 10.1002/jmv.27560 PMC9015237

[31] Du Z, Hong H, Wang S, Ma L, Liu C, Bai Y, et al. Reproduction number of the omicron variant triples that of the delta variant. Viruses. 2022;14(4):821. 35458551 10.3390/v14040821 PMC9027795

[32] Hoffmann M, Kruger N, Schulz S, Cossmann A, Rocha C, Kempf A, et al. The Omicron variant is highly resistant against antibody-mediated neutralization: Implications for control of the COVID-19 pandemic. Cell. 2022;185(3):447-456 e411. 35026151 10.1016/j.cell.2021.12.032 PMC8702401

[33] Uraki R, Kiso M, Iida S, Imai M, Takashita E, Kuroda M, et al. Characterization and antiviral susceptibility of SARS-CoV-2 Omicron/BA.2. Nature. 2022;607(7917):119–27. 35576972 10.1038/s41586-022-04856-1 PMC10579982

[34] Modes ME, Directo MP, Melgar M, Johnson LR, Yang H, Chaudhary P, et al. Clinical Characteristics and Outcomes Among Adults Hospitalized with Laboratory-Confirmed SARS-CoV-2 Infection During Periods of B.1.617.2 (Delta) and B.1.1.529 (Omicron) Variant Predominance - One Hospital, California, July 15-September 23, 2021, and December 21, 2021-January 27, 2022. MMWR Morb Mortal Wkly Rep. 2022;71(6):217–23. 35143466 10.15585/mmwr.mm7106e2 PMC8830624

[35] Wolter N, Jassat W, Walaza S, Welch R, Moultrie H, Groome M, et al. Early assessment of the clinical severity of the SARS-CoV-2 omicron variant in South Africa: a data linkage study. Lancet. 2022;399(10323):437–46. 35065011 10.1016/S0140-6736(22)00017-4 PMC8769664

[36] Li R, Pei S, Chen B, Song Y, Zhang T, Yang W, et al. Substantial undocumented infection facilitates the rapid dissemination of novel coronavirus (SARS-CoV-2). Science. 2020;368(6490):489–93. 32179701 10.1126/science.abb3221 PMC7164387

[37] Li Q, Guan X, Wu P, Wang X, Zhou L, Tong Y, et al. Early transmission dynamics in Wuhan, China, of novel coronavirus-infected pneumonia. N Engl J Med. 2020;382(13):1199–207. 31995857 10.1056/NEJMoa2001316 PMC7121484

[38] Zhu W, Zhang M, Pan J, Yao Y, Wang W. Effects of prolonged incubation period and centralized quarantine on the COVID-19 outbreak in Shijiazhuang, China: a modeling study. BMC Med. 2021;19(1):308. 34872559 10.1186/s12916-021-02178-z PMC8648499

[39] Marks M, Millat-Martinez P, Ouchi D, Roberts CH, Alemany A, Corbacho-Monne M, et al. Transmission of COVID-19 in 282 clusters in Catalonia, Spain: a cohort study. Lancet Infect Dis. 2021;21(5):629–36. 33545090 10.1016/S1473-3099(20)30985-3 PMC7906723

[40] Trobajo-Sanmartin C, Martinez-Baz I, Miqueleiz A, Fernandez-Huerta M, Burgui C, Casado I, et al. : Differences in Transmission between SARS-CoV-2 Alpha (B.1.1.7) and Delta (B.1.617.2) Variants. Microbiology spectrum. 2022;10(2):e0000822. 35412379 10.1128/spectrum.00008-22 PMC9045255

[41] Martinez-Baz I, Trobajo-Sanmartin C, Burgui C, Casado I, Castilla J. Transmission of SARS-CoV-2 infection and risk factors in a cohort of close contacts. Postgrad Med. 2022;134(2):230–8. 35102793 10.1080/00325481.2022.2037360

[42] Reukers DFM, van Boven M, Meijer A, Rots N, Reusken C, Roof I, et al. High Infection Secondary Attack Rates of Severe Acute Respiratory Syndrome Coronavirus 2 in Dutch Households Revealed by Dense Sampling. Clin Infect Dis. 2022;74(1):52–8. 33822007 10.1093/cid/ciab237 PMC8083540

[43] Wu JT, Leung K, Bushman M, Kishore N, Niehus R, de Salazar PM, et al. Estimating clinical severity of COVID-19 from the transmission dynamics in Wuhan. China Nat Med. 2020;26(4):506–10. 32284616 10.1038/s41591-020-0822-7 PMC7094929

[44] Zhong W, Yang X, Jiang X, Duan Z, Wang W, Sun Z, et al. Factors associated with prolonged viral shedding in older patients infected with Omicron BA.2.2. Public Health. 2022;10:1087800. 10.3389/fpubh.2022.1087800 PMC9878338 36711364

[45] Li R, Jin C, Zhang L, Kong D, Hu K, Xuan M, et al. Clinical characteristics and risk factors analysis of viral shedding time in mildly symptomatic and asymptomatic patients with SARS-CoV-2 Omicron variant infection in Shanghai. Front Public Health. 2022;10:1073387. 36684919 10.3389/fpubh.2022.1073387 PMC9845758

[46] Mastrorosa I, Cozzi-Lepri A, Colavita F, Lalle E, Mazzotta V, Cimaglia C, et al. SARS-CoV-2 nasopharyngeal viral load in individuals infected with BA.2, compared to Alpha, Gamma, Delta and BA.1 variants: A single-center comparative analysis. J Clin Virol. 2022;157:105299. 36183546 10.1016/j.jcv.2022.105299 PMC9511897

[47] Li F, Li YY, Liu MJ, Fang LQ, Dean NE, Wong GWK, et al. Household transmission of SARS-CoV-2 and risk factors for susceptibility and infectivity in Wuhan: a retrospective observational study. Lancet Infect Dis. 2021;21(5):617–28. 33476567 10.1016/S1473-3099(20)30981-6 PMC7833912

