# Publisher Correction: Age‑specific transmission dynamics under suppression control measures during SARS‑CoV‑2 Omicron BA.2 epidemic

**DOI:** 10.1186/s12889-026-26853-z

**Published:** 2026-03-24

**Authors:** Wenlong Zhu, Zexuan Wen, Yue Chen, Xiaohuan Gong, Bo Zheng, Xueyao Liang, Ao Xu, Ye Yao, Weibing Wang

**Affiliations:** 1 Shanghai Institute of Infectious Disease and Biosecurity, School of Public Health, Fudan University, 138 Yi Xue Yuan Road, Shanghai, 200032 China; 2 School of Epidemiology and Public Health, University of Ottawa, Ottawa, K1G5Z3 Canada; 3 Institute of Infectious Diseases, Shanghai Municipal Center of Disease Control and Prevention, Shanghai, 200336 China; 4 Key Laboratory of Public Health Safety of Ministry of Education, Fudan University, 138 Yi Xue Yuan Road, Shanghai, 200032 China

**Publisher Correction: BMC Public Health 23, 743 (2023)**

**https://doi.org/10.1186/s12889-023-15596-w**

After correspondence between the Journal and the authors of this article [1], the Journal determined that the COVID-19 epidemic data for Wuhan is not available for public dissemination. This data has not been shared with the Journal. This article’s data availability statement, which originally read “Location data of each case reported before 17 March and codes used during the current study are available from the corresponding author on reasonable request.”, has therefore been amended to “The COVID-19 epidemic data for Wuhan is sequestered and cannot be publicly shared.”
