# Supplementary material for: Age-specific transmission dynamics under suppression control measures during SARS-CoV-2 Omicron BA.2 epidemic
Source: BMC Public Health. 2023 Apr 22;23:743. doi: 10.1186/s12889-023-15596-w (PMC10121427; doi:10.1186/s12889-023-15596-w)
Supplement: Supplementary file 1 — Additional file 1: Table S1. Meanings and initial values of parameters in the age-structured SEIR model. Table S2. Outcomes, parameters and their values used in sensitivity analysis. Figure S1. Patterns of parameters (p_E, p_NAAT, and p_t) variation with date. A: Proportion of untraced exposed persons (p_E). B: Proportion of cases found by NAAT/RAT (p_NAAT). C: Proportion of concentrated isolation among positive NAAT cases (p_t). Figure S2. Values and regularity of p_E (A) and p_NAAT (B) used in sensitivity analysis. Figure S3. Cumulative number of cases in different districts of Shanghai. Figure S4. The potential areas of virus had been spread on different date of the epidemics in Shanghai (A) and Wuhan (B) with different mean transmission distance (namely the radius of each circle: 13.4 km in Shanghai and 4.4 km in Wuhan). The cumulative number of cases was same for each column. Figure S5. Estimated contract matrices at different settings over seven periods in Shanghai. Figure S6. Demographic comparison of population (A), cases (B) and standard incidence (C) for Shanghai and Wuhan. Figure S7. Effective reproductive number (Rt) in the epidemic of Shanghai. Rt was estimated from4-day and 7-day moving average respectively. Figure S8. Epidemic curve of cumulative predicted cases and reported cases estimate by the model with different values of parameters. Figure S9. The proportion of unascertained cases under different proportion of asymptomatic cases (A), proportion of untraced cases (B) and proportion of cases detected by NAAT/RAT (C). Figure S10. Comparation of estimated secondary attack rates (SAR) and estimated reported SAR (SARobs). Blue and red dashed line is overall SAR (24.1%) and overall SARobs (17.4%). [file 12889_2023_15596_MOESM1_ESM.docx]

**Supplemental Materials for**

**Age-specific transmission dynamics under suppression control measures during SARS-CoV-2 Omicron BA.2 epidemic**

Wenlong Zhu^1†^, Zexuan Wen^1†^, Yue Chen^2^, Xiaohuan Gong^3^, Bo Zheng^1^, Xueyao Liang^1^, Ao Xu^1^, Ye Yao^1^*, Weibing Wang^1,4^*

^1^School of Public Health, Shanghai Institute of Infectious Disease and Biosecurity, Fudan University, Shanghai 200032, China

^2^School of Epidemiology and Public Health, University of Ottawa, Ottawa K1G5Z3, Canada

^3^Institute of Infectious Diseases, Shanghai Municipal Center of Disease Control and Prevention, Shanghai 200336, China

^4^Key Laboratory of Public Health Safety of Ministry of Education, Fudan University, Shanghai 200032, China

† These authors contributed equally to this work.

* Corresponding:

Dr. Ye Yao, Department of Biostatics, School of Public Health, Fudan University, 138 Yi Xue Yuan Road, Shanghai 200032, China (e-mail: yyao@fudan.edu.cn).

Dr. Weibing Wang, Department of Epidemiology, School of Public Health, Fudan University, 138 Yi Xue Yuan Road, Shanghai 200032, China (e-mail: wwb@fudan.edu.cn).

**This file includes:**

Supplementary methods:

*Role of vaccination*

*Constructing social contact matrix*

*Model calibration*

*Next generation matrix*

*Inferring the susceptibility and infectivity*

*Sensitivity analysis*

Table S1 to Table S2.

Figure S1 to Figure S10.

**Other supplementary materials:**

Movies S1. Areas with infection risk in the Omicron epidemic of Shanghai form 26 February to 17 March 2022. The radius of each circle (13.4 km) is the mean transmission distances between any of case-pairs.

**Supplementary** **methods**

***Role of vaccination***

In the model, the age-specific vaccination coverage was used to rescaling the susceptible population. Coverage ($p_{v2}^{i}$, $p_{v3}^{i}$) and effectiveness (${Ve}_{2}$, ${Ve}_{3}$) of the second and third doses vaccines against Omicron infection were considered in the model, since one dose is not effective [18]. Few COVID-19 cases were reported in Shanghai before 20th February 2022 [15]. Thus, natural and hybrid immunity were not considered in this model. Data on age-specific coverage of the second and third doses vaccines on 15th April 2022 were used in the analysis, and we assumed that vaccination coverage were constant over the study period (20th February to 15th June 2022). Vaccination waning was neglected, the vaccination effectiveness against infection of the second and third doses vaccines kept constant and obtained from Huang Z. et al [18] and Palacios, R. et al [19]. The vaccination effectiveness against severe/critical illness, hospitalization and death were not considered in this study.

***Constructing social contact matrix***

Since 1 March, residents lived in the same residential districts where reported COVID-19 cases were asked to quarantine at home. On 12th March, all schools were closed. Public transplantation had been suspended gradually since 14th March. Home quarantine and NAAT were carried out in Chongming, Fengxian, Jinshan, Minhang, Pudong and Songjiang starting 28th March, and the rest 10 districts starting on 4th April. Since 5th April, all public transportation had been suspended, and all unessential inner-city travels were prohibited.

In the SEIR model, we considered the age-specific contact matrices ($CM$) from previous studies to capture individual human interactions approximately. The contact pattern before the epidemic (namely, the normal social contacts) was based on data from a survey conducted between December 2017 and May 2018 in Shanghai [20]. The above interventions had a slightly impact on the number of contacts at home, but reduced the number of contacts at other locations (such as schools and workplaces) [21]. In our model, the number of contacts at school was zero since 12 March, and the decline of the number of contacts at workplaces and other places was assumed to be consistent with the decrease of mobility in Shanghai.

Based on the age-specific (0-15, 16-17, 18-29, 30-39, 40-49, 0-59 and 60+) mobile phone location data provided by the SmartSteps (http://www.smartsteps.com/), we calculated the percentage of decline in mobility over six periods (7 to 13 March, 14 to 20 March, 21 to 27 March, 28 March to 3 April, 4 to 10 April and since 11 April) compared to the normal period (before 6 March). Then, we constructed the contact matrices at workplaces and other places over the six periods. The number of contacts at home (${CM}_{H}$) was assumed to be consistent over the seven periods. The number of contacts at school before 12 March was consistent with the normal period but decreased to zero after the school closure since 12 March. These contact matrices were used in the age-structured SEIR model.

***Model calibration***

Our age-structured model could be described in the equations below.

$$p_{nv}^{i}=1-\left( p_{v2}^{i}-p_{v3}^{i} \right)*{Ve}_{2}-p_{v3}^{i}*{Ve}_{3}$$

$$\frac{{dS}^{i}}{dt}=-\frac{\beta^{i}*S^{i}*p_{nv}^{i}*\left[ CM*\left( \varepsilon A^{i}+{\varepsilon P}^{i}+I^{i} \right)+{CM}_{H}*\left( \varepsilon A_{H}^{i}+{\varepsilon P}_{H}^{i} \right) \right]}{N^{i}}$$

$$\frac{{dE}^{i}}{dt}=\frac{\beta^{i}*S^{i}*p_{nv}^{i}*\left[ p_{E}*CM*\left( \varepsilon A^{i}+{\varepsilon P}^{i}+I^{i} \right)+{CM}_{H}*\left( \varepsilon A_{H}^{i}+{\varepsilon P}_{H}^{i} \right) \right]}{N^{i}}-\frac{E^{i}}{\alpha}$$

$$\frac{{dA}^{i}}{dt}=\frac{p_{A}*E^{i}}{\alpha}-\frac{{{(1-p}_{NAAT})*A}^{i}}{\gamma_{A}}-\frac{{{p_{t}*p}_{NAAT}*A}^{i}}{\alpha_{3}}-{(1-{p_{t})*p}_{NAAT}*A}^{i}$$

$$\frac{{dA}_{H}^{i}}{dt}={(1-{p_{t})*p}_{NAAT}*A}^{i}-\frac{A_{H}^{i}}{\alpha_{H}}$$

$$\frac{{dP}^{i}}{dt}=\frac{{(1-p}_{A})*E^{i}}{\alpha}-\frac{{{(1-p}_{NAAT})*P}^{i}}{\alpha_{2}}-\frac{{{p_{t}*p}_{NAAT}*P}^{i}}{\alpha_{3}}-{{(1-p_{t})*p}_{NAAT}*P}^{i}$$

$$\frac{{dP}_{H}^{i}}{dt}={(1-{p_{t})*p}_{NAAT}*P}^{i}-\frac{P_{H}^{i}}{\alpha_{H}}$$

$$\frac{{dI}^{i}}{dt}=\frac{{{(1-p}_{NAAT})*P}^{i}}{\alpha_{2}}-\frac{I^{i}}{\alpha_{4}}$$

$$\frac{{dE}_{Q}^{i}}{dt}=\frac{(1-{p_{E})*\beta}^{i}*S^{i}*p_{nv}^{i}*CM*\left( \varepsilon A^{i}+{\varepsilon P}^{i}+I^{i} \right)}{N^{i}}-\frac{E_{Q}^{i}}{\alpha}$$

$$\frac{{dA}_{Q}^{i}}{dt}=\frac{p_{t}*{p_{NAAT}*A}^{i}}{\alpha_{3}}+\frac{p_{A}*E_{Q}^{i}}{\alpha}+\frac{A_{H}^{i}}{\alpha_{H}}-\frac{A_{Q}^{i}}{\gamma_{A}}$$

$$\frac{{dP}_{Q}^{i}}{dt}=\frac{p_{t}*{p_{NAAT}*P}^{i}}{\alpha_{3}}+\frac{{(1-p}_{A})*E_{Q}^{i}}{\alpha}-\frac{P_{Q}^{i}}{\alpha_{2}}$$

$$\frac{{dI}_{Q}^{i}}{dt}=\frac{I^{i}}{\alpha_{4}}+\frac{P_{Q}^{i}}{\alpha_{2}}+\frac{P_{H}^{i}}{\alpha_{H}}-\frac{I_{Q}^{i}}{\gamma_{I}}$$

$$\frac{{dR}^{i}}{dt}=\frac{{{(1-p}_{NAAT})*A}^{i}+A_{Q}^{i}}{\gamma_{A}}+\frac{I_{Q}^{i}}{\gamma_{I}}$$

With the number of daily reported cases, the least square method (LSM) was used to calibrate the model and to estimate the age-specific transmission rate ($\beta^{i}$). The transmission rate with a minimized residual sum of squares ($RSS$) was the best estimation. $X_{obs,j}^{i}$ is the number of reported cases of $i$ age group on $j$ day. The cumulative reported cases were related to another compartment $\frac{{dCf}^{i}}{dt}$. $X_{sim,j}^{i}$ was the difference between the cumulative reported infections of $i$ age group on $j$ day (${Cf}_{j}^{i}$) and $j-1$ day (${Cf}_{j-1}^{i}$). A parametric bootstrap approach was used to explore uncertainty in the estimation of $\beta^{i}$.

$$RSS=\sum_{i}^{10} \sum_{j}^{n} \left( X_{sim, j}^{i}-X_{obs,j}^{i} \right)^{2}$$

$$\frac{{dCf}^{i}}{dt}=\frac{E_{Q}^{i}}{\alpha}+\frac{p_{t}*{p_{NAAT}*(A}^{i}+P^{i})}{\alpha_{3}}+\frac{\left( A_{H}^{i}+P_{H}^{i} \right)}{\alpha_{H}}+\frac{I^{i}}{\alpha_{4}}$$

$$X_{sim, j}^{i}={Cf}_{j}^{i}-{Cf}_{j-1}^{i}$$

$$i \left( age groups \right)=1,2,\ldots\ldots,10;j \left( days \right)=1, 2, \ldots\ldots,n$$

***Next generation matrix***

The next generation matrix (NGM) was used to estimate the basic reproduction number (R_0_) [24-26]. We go through the method here for our model. NGM consists transmission part ($F$) and transition part ($V$). $F$ describes the production of new infections, and $V$ describes changes in state. Thus, the NGM is equal to $-FV^{-1}$.

In our model, the $F$ matrix can be expressed as a block matrix, and each block ($F_{mn}$) corresponds to an age group.

$$F=\left[ \begin{matrix} \begin{matrix} F_{11} & F_{12} \\ F_{21} & F_{22} \end{matrix} & \cdots& \begin{matrix} F_{1i} \\ F_{2i} \end{matrix} \\ \vdots& \ddots& \vdots\\ \begin{matrix} F_{i1} & F_{i2} \end{matrix} & \cdots& F_{ii} \end{matrix} \right], i \left( age groups \right)=1,2,\ldots\ldots,10$$

$$F_{mn}=\left[ \begin{matrix} 0 & p_{E}*\varepsilon*{CM}_{mn}*f_{mn} & p_{E}*\varepsilon*{CM}_{mn}*f_{mn} & p_{E}*{CM}_{mn}*f_{mn} & \varepsilon*{CM}_{Hmn}*f_{mn} & \varepsilon*{CM}_{Hmn}*f_{mn} \\ 0 & 0 & 0 & 0 & 0 & 0 \\ 0 & 0 & 0 & 0 & 0 & 0 \\ 0 & 0 & 0 & 0 & 0 & 0 \\ 0 & 0 & 0 & 0 & 0 & 0 \\ 0 & 0 & 0 & 0 & 0 & 0 \end{matrix} \right]$$

$$f_{mn}=\beta_{m}*\left( 1-\left( p_{v2}^{n}-p_{v3}^{n} \right)*{Ve}_{2}-p_{v3}^{n}*{Ve}_{3} \right)*\frac{S_{m}}{N_{n}}$$

It is worth noting that $m$ and $n$ in $F_{mn}$ and $f_{mn}$ correspond to the block indices of $F$, not their cell position in the matrix.

We assumed that there was no movement between the age groups. Thus, the $V$ matrix is expressed as a block diagonal matrix where each block corresponds to an age group.

$$V=\left[ \begin{matrix} \begin{matrix} V_{11} & V_{12} \\ V_{21} & V_{22} \end{matrix} & \cdots& \begin{matrix} V_{1i} \\ V_{2i} \end{matrix} \\ \vdots& \ddots& \vdots\\ \begin{matrix} V_{i1} & V_{i2} \end{matrix} & \cdots& V_{ii} \end{matrix} \right]$$

$$V_{ii}=\left[ \begin{matrix} -\frac{1}{\alpha} & 0 & 0 & 0 & 0 & 0 \\ \frac{p_{A}}{\alpha} & -\frac{1-p_{NAAT}}{\gamma_{A}}-\frac{p_{t}*p_{NAAT}}{\alpha_{3}}-\left( 1-p_{t} \right)*p_{NAAT} & 0 & 0 & 0 & 0 \\ \frac{1-p_{A}}{\alpha} & 0 & -\frac{1-p_{NAAT}}{\alpha_{2}}-\frac{p_{t}*p_{NAAT}}{\alpha_{3}}-\left( 1-p_{t} \right)*p_{NAAT} & 0 & 0 & 0 \\ 0 & 0 & \frac{1-p_{NAAT}}{\alpha_{2}} & -\frac{1}{\alpha_{4}} & 0 & 0 \\ 0 & \left( 1-p_{t} \right)*p_{NAAT} & 0 & 0 & -\frac{1}{\alpha_{H}} & 0 \\ 0 & 0 & \left( 1-p_{t} \right)*p_{NAAT} & 0 & 0 & -\frac{1}{\alpha_{H}} \end{matrix} \right]$$

The dominant eigenvalue of NGM ($-FV^{-1}$) was the basic reproduction number (R_0_).

***Inferring the susceptibility and infectivity***

To calculate the secondary attack rate (SAR), an additional compartment that count the total contacts of each age group is needed. The total number of contacts in each age group were calculated according to the following equation. Combined with the cumulative number of ascertained cases (${Cf}^{i}$) and all cases ($C^{i}$, ascertained and unascertained) in each age group, the estimated reported SAR (${SAR}_{obs}^{i}$) and estimated SAR (${SAR}^{i}$) could be calculated respectively.

$$\frac{{dContact}^{i}}{dt}=\frac{S^{i}*p_{nv}^{i}*\left[ CM*\left( \frac{\varepsilon*E^{i}}{\alpha}+\frac{{{(1-p}_{NAAT})*P}^{i}}{\alpha_{2}} \right)+{CM}_{H}*\varepsilon*\left( 1-p_{t} \right)*p_{NAAT}*\left( A^{i}+P^{i} \right) \right]}{N^{i}}$$

${SAR}_{obs}^{i}={Cf}^{i}/{Contact}^{i}$, ${SAR}^{i}=C^{i}/{Contact}^{i}$

With the $\beta^{i}$and estimated SAR (${SAR}^{i}$), we inferred the susceptibility and infectivity and tried to prioritize different age groups for centralized isolation and NAAT/RAT. Reference scenario in analyzing priority of isolation is that all cases of different ages were self-isolated at home. Other scenarios consider that cases in one of the age groups were centralized isolated, while cases in the other age groups remained self-isolated at home. The number of secondary cases infected by index cases self-isolated at home are calculated and compared. The less of the number of cases infected by index cases, the higher the priority of centralized isolation should be given to implementing centralized isolation in these age groups. The reference scenario in analyzing priority of NAAT/RAT is that NAAT/RAT are implemented in cases of all ages. The cumulative number of cases under lifting NAAT/RAT in different age groups is simulated and compared with that of reference scenario. The more the cumulative number of cases, the greater the impact of these age groups, and priority should be given to implementing NAAT/RAT in these age groups.

***Sensitivity analysis***

In this part, we explored the impacts of the relative transmissibility rate of asymptomatic cases to symptomatic cases ($\varepsilon$), proportion of asymptomatic cases ($p_{A}$), period of self-isolation at home ($\alpha_{H}$) and recovery period of asymptomatic cases ($\gamma_{A}$) on the transmission rates ($\beta^{i}$), proportion of cases found by NAAT/RAT ($p_{NAAT}$), secondary attack rates (${SAR}^{i}$), cumulative incidences, basic reproduction number (R_0_) and proportion of unascertained cases (Table S2). New values of these parameters were used to recalibrate the age-structed SEIR model and simulate the other results to explore the robustness of this model and results.

Additionally, we accessed the impacts of proportion of untraced cases ($p_{E}$), proportion of asymptomatic cases ($p_{A}$) and proportion of cases found by NAAT/RAT ($p_{NAAT}$) on the proportion of unascertained cases by sensitivity analysis. In this analysis (SA2), the model used to simulate was calibrated base on the parameters in Table S1. Impacts of $p_{A}$ that value changed from 0 to 1 on the proportion of unascertained cases was assessed. In the sensitivity analysis, the changing regularity of $p_{E}$ and $p_{NAAT}$ was the same of Figure S1, but the values at each time point were multiplied by a scaling parameter (0.5 to 1.5). The maximal value of $p_{E}$ and $p_{NAAT}$ was 1. An example of values of $p_{E}$ and $p_{NAAT}$ used in sensitivity analysis was presented in Figure S2.

**Tables**

**Table S1. Meanings and initial values of parameters in the age-structured SEIR model.**

| **Parameters** | **Meaning** | **Values** | **Sources** |
| --- | --- | --- | --- |
| $S^{i}$ | Susceptible population | [0, 3): 454600  [3, 12): 1610700  [12, 18): 714700  [18, 30): 4086500  [30, 40): 5037100  [40, 50): 3633800  [50, 60): 3518100  [60, 70): 3414100  [70, 80): 1565600  80+: 835900 | Shanghai Statistical year book 2021 [14] |
| $E^{i}$ | Exposed population | 0 for all age groups | Ref [15] |
| $A^{i}$ | Asymptomatic infections | There were 2 asymptomatic infections in the [40, 50) year old group and the other groups were assumed to be 0. |  |
| $A_{H}^{i}$ | Asymptomatic infections that are self-isolated at home | 0 for all age groups |  |
| $P^{i}$ | Presymptomatic infections | 0 for all age groups |  |
| $P_{H}^{i}$ | Presymptomatic infections that are self-isolated at home | 0 for all age groups |  |
| $I^{i}$ | Symptomatic cases | 0 for all age groups |  |
| $E_{Q}^{i}$ | Exposed population that are concentrated isolated | There were 16 concentrated isolated exposed inidividuals in the [40, 50) year old group and the other groups were assumed to be 0. |  |
| $A_{Q}^{i}$ | Asymptomatic infections that are concentrated isolated | 0 for all age groups |  |
| $P_{Q}^{i}$ | Presymptomatic infections that are concentrated isolated | 0 for all age groups |  |
| $I_{Q}^{i}$ | Symptomatic cases that are concentrated isolated | 0 for all age groups |  |
| $R^{i}$ | Removed population, including recovered and death | 0 for all age groups |  |
| $\beta^{i}$ | Transmission rate of different age groups (i) | [0, 3): 0.008 (95% CI: 0.007 to 0.009)  [3, 12): 0.049 (95% CI: 0.047 to 0.050)  [12, 18): 0.031 (95% CI: 0.030 to 0.032)  [18, 30): 0.122 (95% CI: 0.117 to 0.126)  [30, 40): 0.162 (95% CI: 0.160 to 0.175)  [40, 50): 0.195 (95% CI: 0.192 to 0.198)  [50, 60): 0.216 (95% CI: 0.210 to 0.222)  [60, 70): 0.137 (95% CI: 0.133 to 0.141)  [70, 80): 0.079 (95% CI: 0.076 to 0.081)  80+: 0.038 (95% CI: 0.037 to 0.040) | Estimated |
| $\varepsilon$ | Relative transmissibility rate of asymptomatic cases to symptomatic cases | 0.6 | Ref [16] |
| $\alpha$ | Latent period | 2 days | Ref [17] |
| $\alpha_{2}$ | Period of presymptomatic cases show symptoms (Incubation period-Latent period) | 1.2 days, incubation period = 3.2 days | Ref [17] |
| $\alpha_{3}$ | Period between positive NAAT and isolation among asymptomatic and presymptomatic cases | 1 day | Assumed |
| $\alpha_{4}$ | Period between symptom onset and isolation among symptomatic cases | 2 days | Assumed |
| $\alpha_{H}$ | Period of self-isolation at home | 2 days | Assumed |
| $\gamma_{A}$ | **Recovery period of asymptomatic cases** | 5 days | Assumed |
| $p_{E}$^*^ | Proportion of untraced exposed persons | Decreased from 1 to 0 gradually. | Assumed |
| $p_{A}$ | Proportion of asymptomatic cases | 0.9 | Calculated |
| $p_{NAAT}$^*^ | Proportion of cases found by NAAT/RAT | Before 28 March:  0.120 (95% CI: 0.115 to 0.125)  28 March to 4 April:  0.168 (95% CI: 0.160 to 0.176)  5 to 21 April:  0.292 (95% CI: 0.286 to 0.299)  After 21 April:  0.490 (95% CI: 0.478 to 0.503) | Estimated |
| $p_{t}$^*^ | Proportion of concentrated isolation among positive NAAT cases | Before 28 March: 1; 28 March to 12 April: 0.54 on 28 March, gradually increased to 1; After 12 April: 1 | Assumed |

* $p_{E}$, $p_{NAAT}$, and $p_{t}$ are parameters that vary with date as presented in Figure S1.

**Table S2. Outcomes, parameters and their values used in sensitivity analysis.**

| **Group** | **Parameters in sensitivity analysis** | **Value** | **Other parameters** | **Outcomes** |
| --- | --- | --- | --- | --- |
| SA1 | Relative transmissibility rate of asymptomatic cases to symptomatic cases ($\varepsilon$) | 0.3, 0.9 | The same in Table S1 | Model calibration:  Transmission rates ($\beta^{i}$)  Proportion of cases found by NAAT/RAT ($p_{NAAT}$)  Model simulation:  Secondary attack rates  Cumulative incidences  Basic reproduction number  Proportion of unascertained cases |
|  | Proportion of asymptomatic cases ($p_{A}$) | 0.3, 0.6 |  |  |
|  | Period of self-isolation at home ($\alpha_{H}$) | 4 days, 6 days |  |  |
|  | Recovery period of asymptomatic cases ($\gamma_{A}$) | 3 days, 7 days |  |  |
| SA2 | Proportion of untraced cases ($p_{E}$) | 0.5 to 1.5 (difference=0.1) times of initial value, maximal value=1 | The same in Table S1 | Proportion of unascertained cases |
|  | Proportion of asymptomatic cases ($p_{A}$) | 0 to 1, difference=0.1 |  |  |
|  | Proportion of cases found by NAAT/RAT ($p_{NAAT}$) | 0.5 to 1.5 (difference=0.1) times of initial value, maximal value=1 |  |  |

**Figures**

**
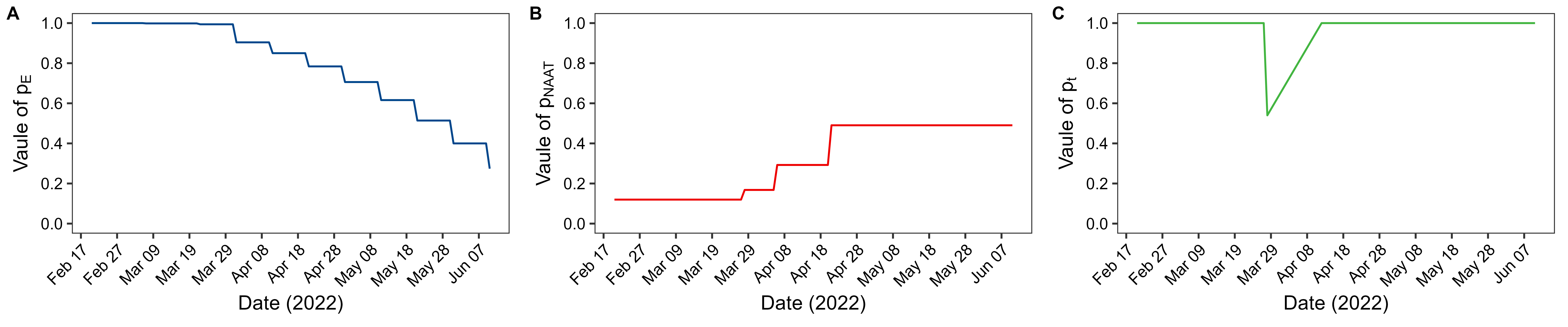
****Figure S1. Patterns of parameters (**$\boldsymbol{p}_{\boldsymbol{E}}$**,** $\boldsymbol{p}_{\boldsymbol{NAAT}}$**, and** $\boldsymbol{p}_{\boldsymbol{t}}$**) variation with date.** **A**: Proportion of untraced exposed persons ($p_{E}$). **B**: Proportion of cases found by NAAT/RAT ($p_{NAAT}$). **C**: Proportion of concentrated isolation among positive NAAT cases ($p_{t}$).

**
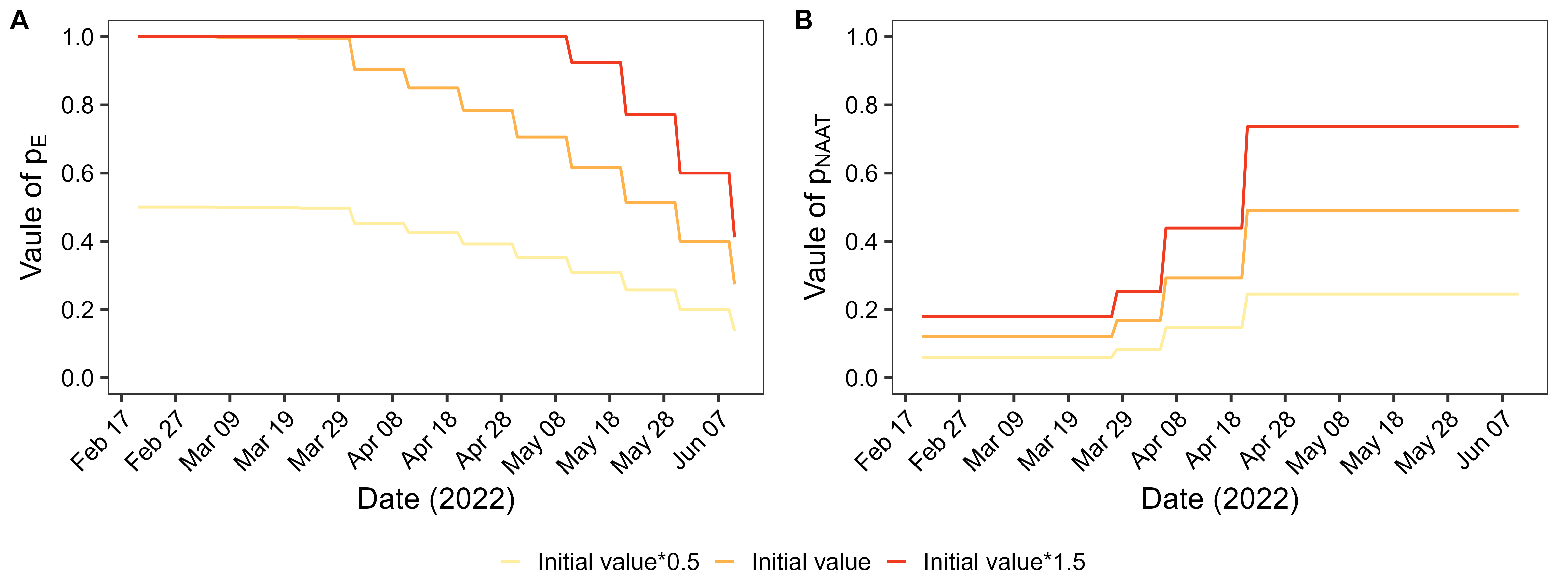
Figure S2. Values and regularity of** $\boldsymbol{p}_{\boldsymbol{E}}$ **(A) and** $\boldsymbol{p}_{\boldsymbol{NAAT}}$ **(B) used in sensitivity analysis.**

**Figure S3. Cumulative number of cases in different districts of Shanghai.** Central areas were: ① Yangpu, ② Hongkou, ③ Jing’an, ④ Putuo, ⑤ Changning, ⑥ Huangpu.
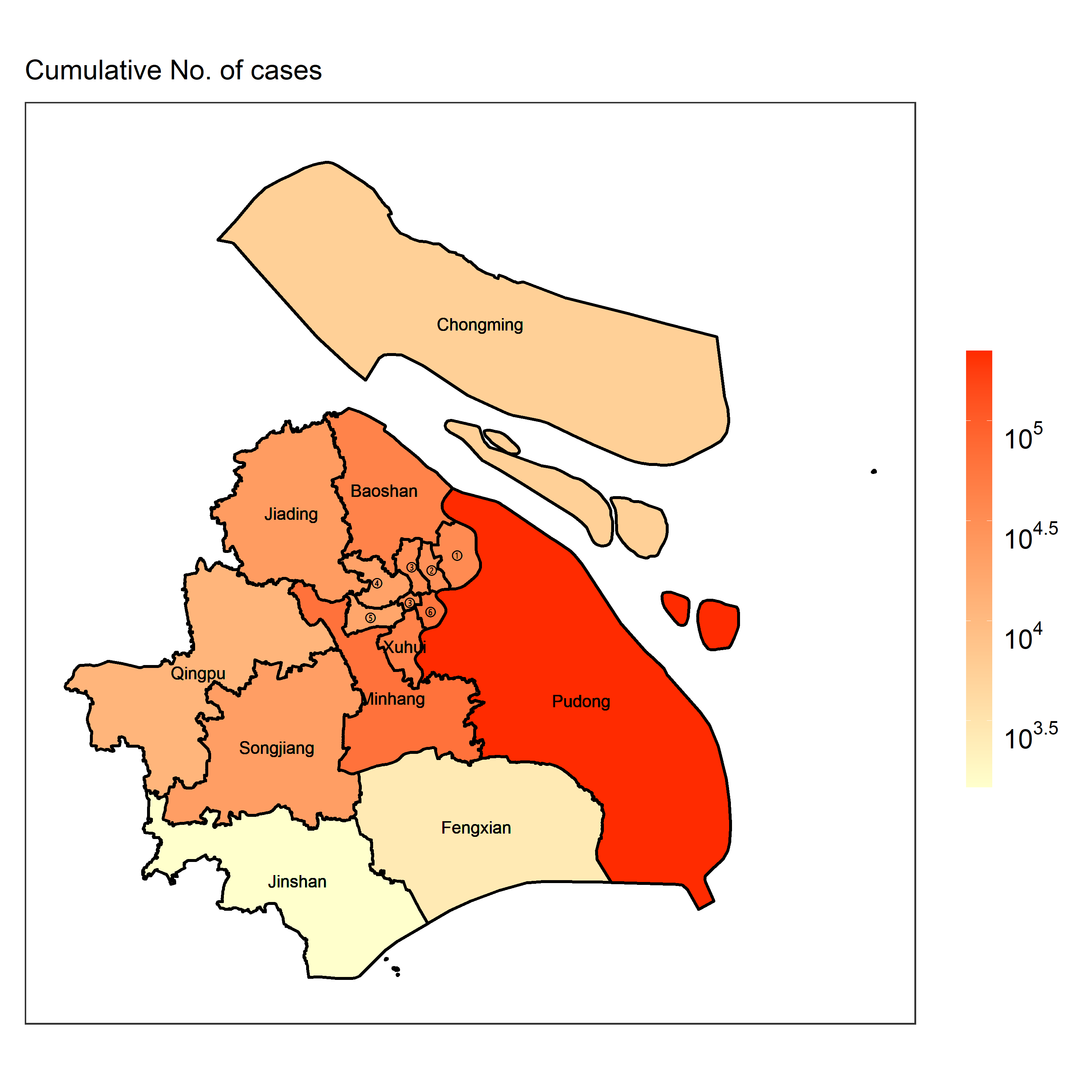


**
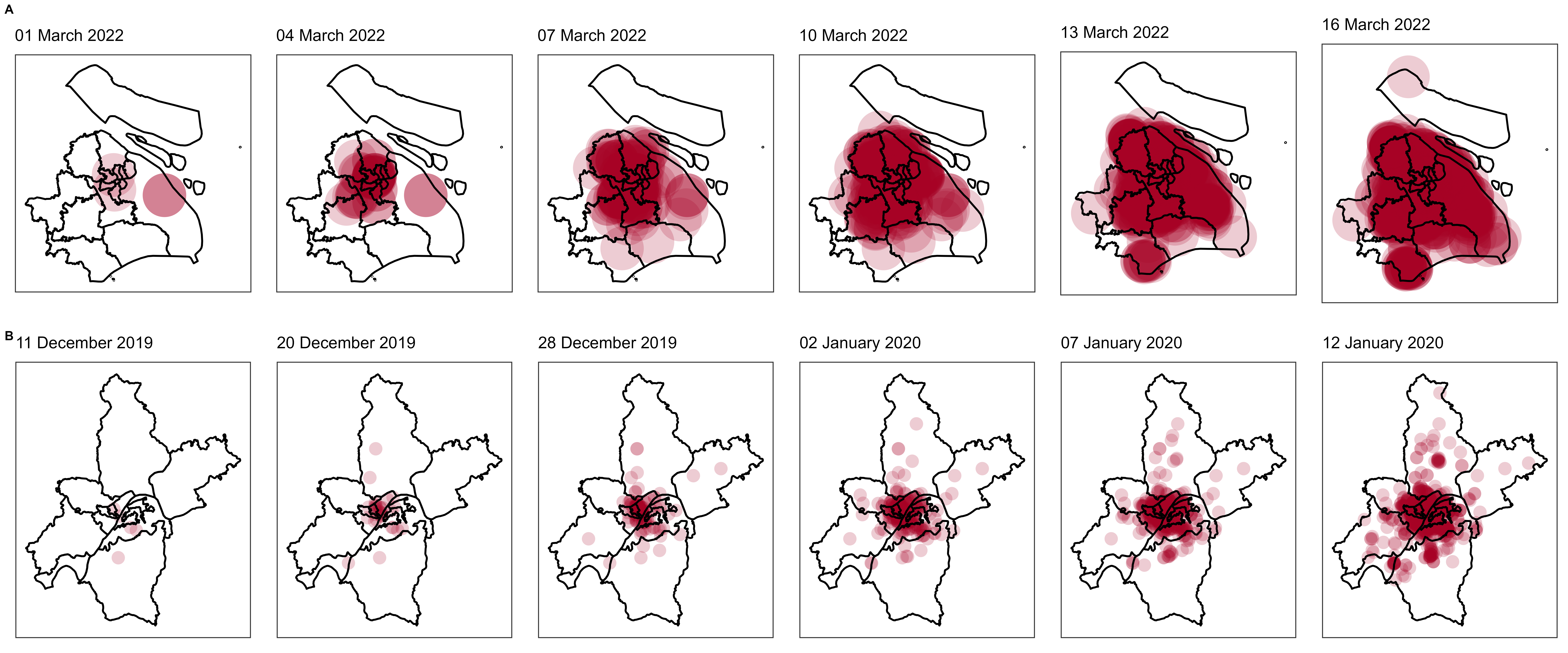
Figure S4. The potential areas of virus had been spread on different date of the epidemics in Shanghai (A) and Wuhan (B) with different mean transmission distance (namely the radius of each circle: 13.4 km in Shanghai and 4.4 km in Wuhan).** The cumulative number of cases was same for each column.

**
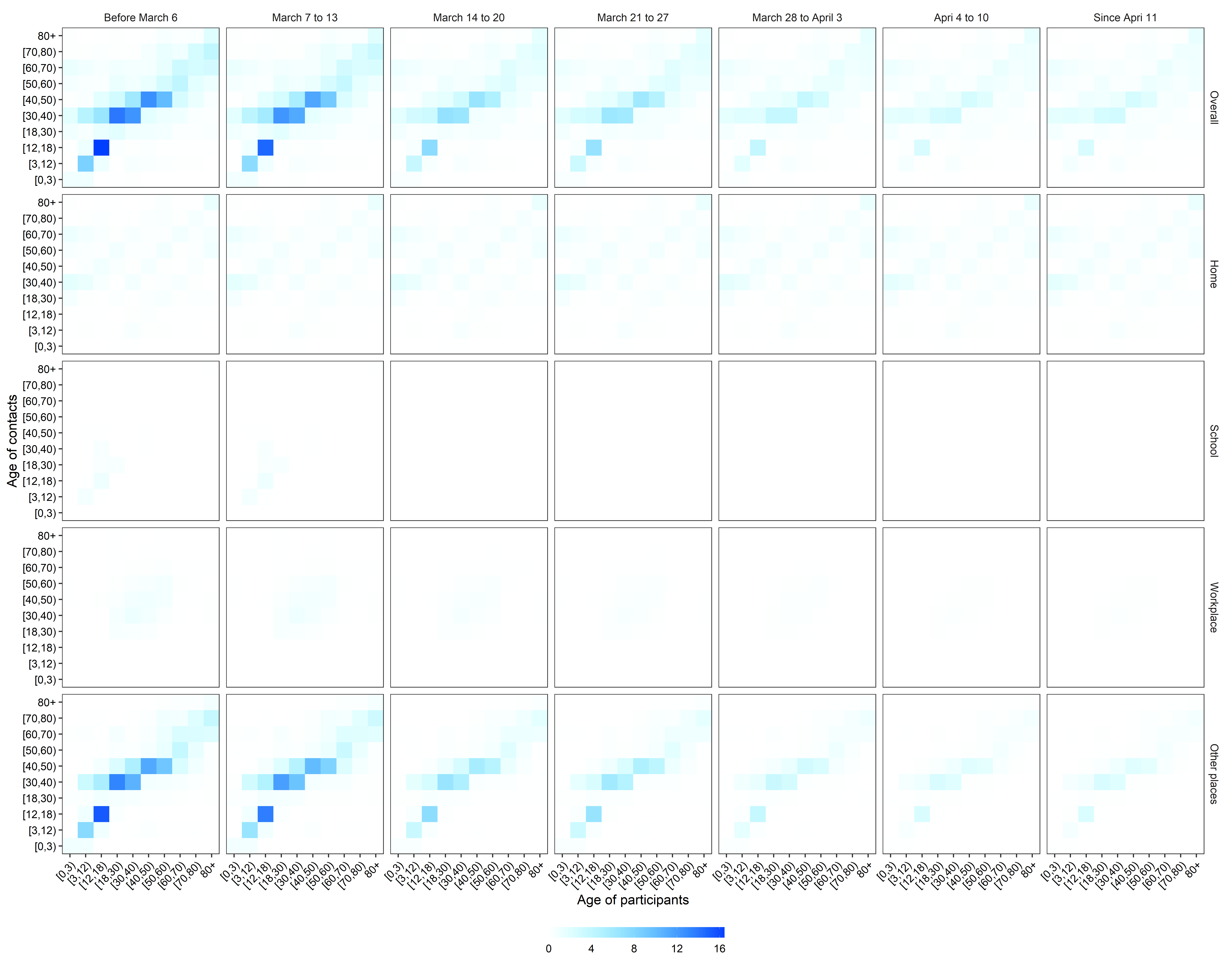
Figure S5. Estimated contract matrices at different settings over seven periods in Shanghai.** The contact pattern before the epidemic (namely, the normal social contacts) was based on data from a survey conducted between December 2017 and May 2018 in Shanghai [20]. Interventions (school closure, suspension of public transportation and home quarantine) implemented in Shanghai have slightly impact on the number of contacts at home, while reduce the number of contacts at other locations (such as schools and workplaces) [21]. The number of contacts at school was zero since 12 March, and the decline of the number of contacts at workplaces and other places was assumed to be consistent with the decrease of mobility in Shanghai.

**
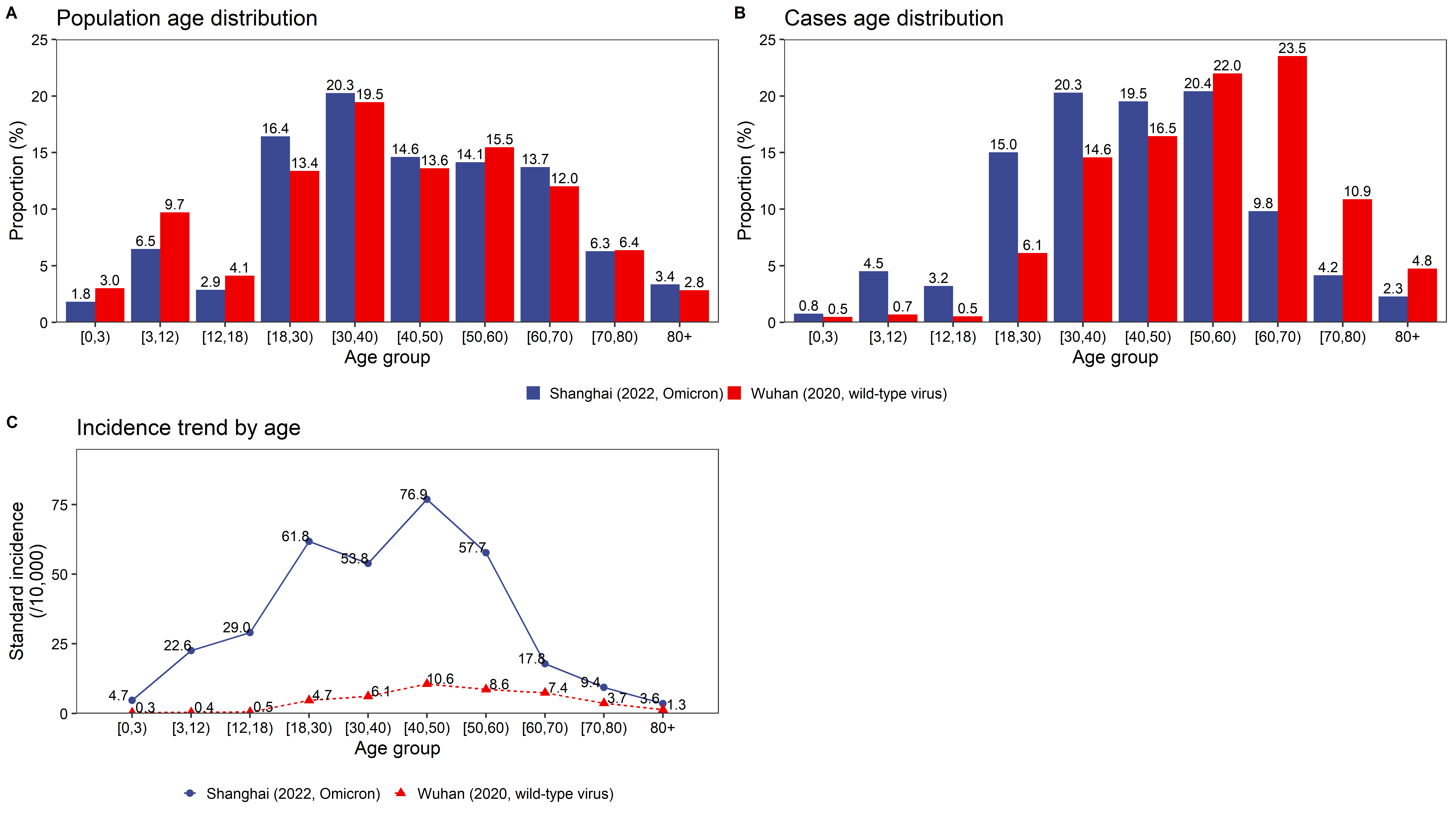
Figure S6. Demographic comparison of population (A), cases (B) and standard incidence (C) for Shanghai and Wuhan.** In **B** The number of cases in different ages in Shanghai were estimated form the SEIR model, and the number of cases in different ages in Wuhan were derived from previous studies [27, 28]. Age distribution of standard population used in **C** were from the 2010 population census of China.

**
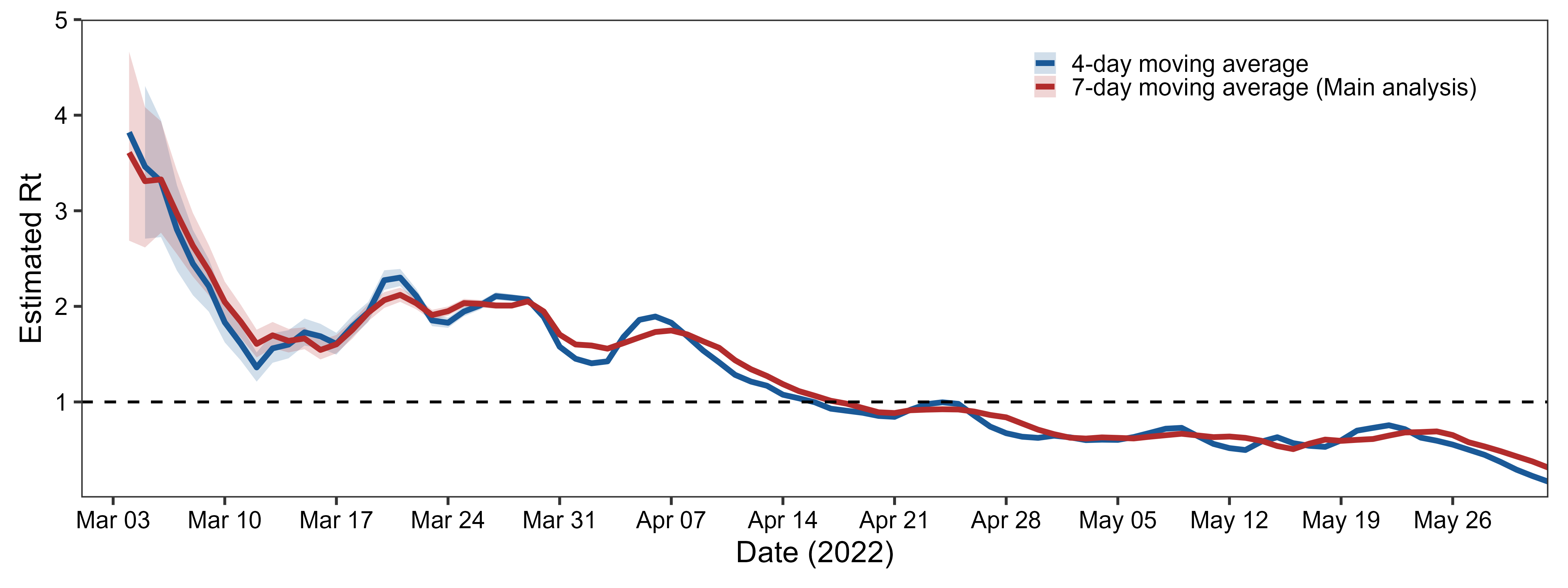
Figure S7. Effective reproductive number (R_t_) in the epidemic of Shanghai.** R_t_ was estimated from4-day and 7-day moving average respectively.

**
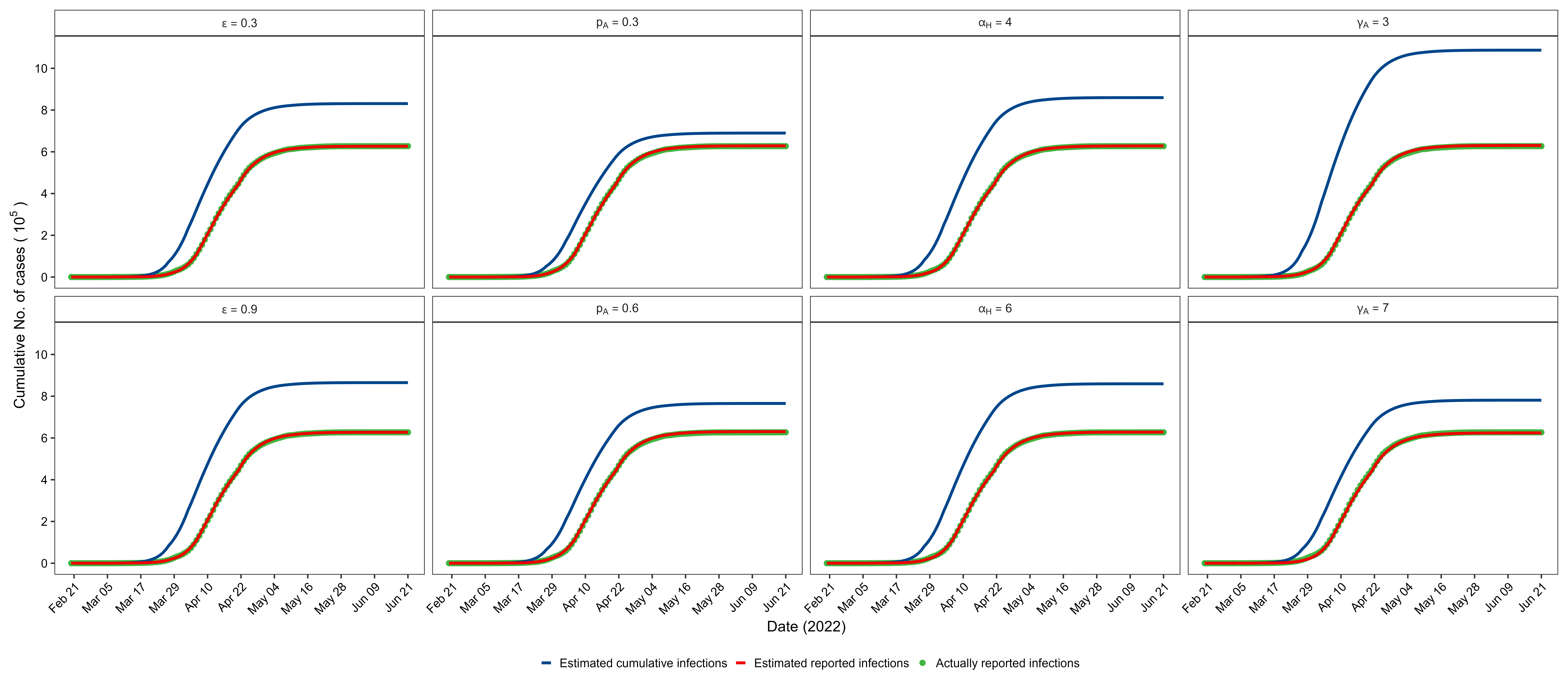
Figure S8. Epidemic curve of cumulative predicted cases and reported cases estimate by the model with different values of parameters.** $\varepsilon$: Relative transmissibility rate of asymptomatic cases to symptomatic cases; $p_{A}$: Proportion of asymptomatic cases; $\alpha_{H}$: Period of self-isolation at home; $\gamma_{A}$: recovery period of asymptomatic cases. Values of parameters used in calibration and simulation were presented in Table S2.


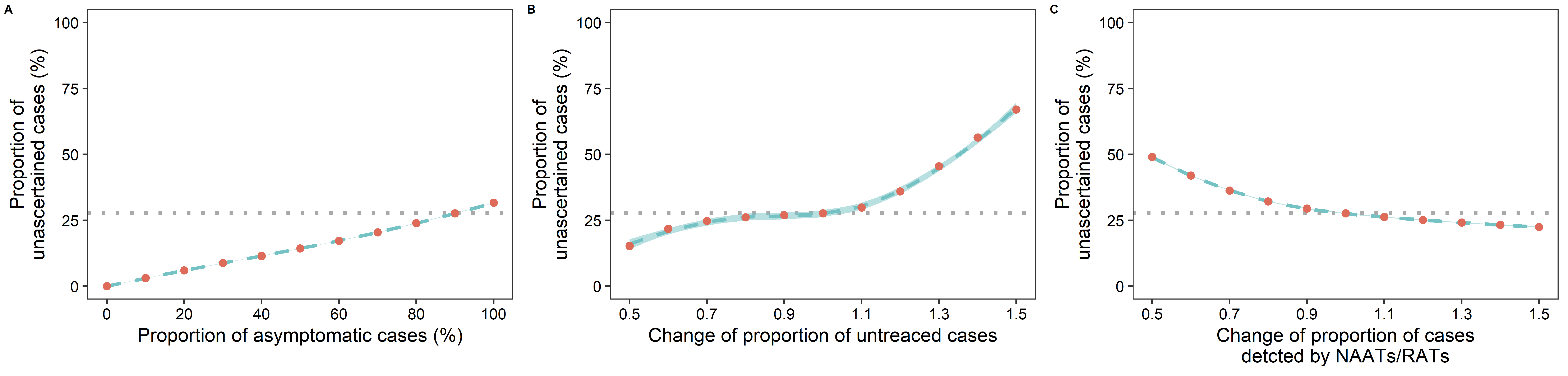


**Figure S9. The proportion of unascertained cases under different proportion of asymptomatic cases (A), proportion of untraced cases (B) and proportion of cases detected by NAAT/RAT (C).** The dotted grey lines are the proportion of unascertained cases (27.7%) in the Omicron epidemic of Shanghai.

**
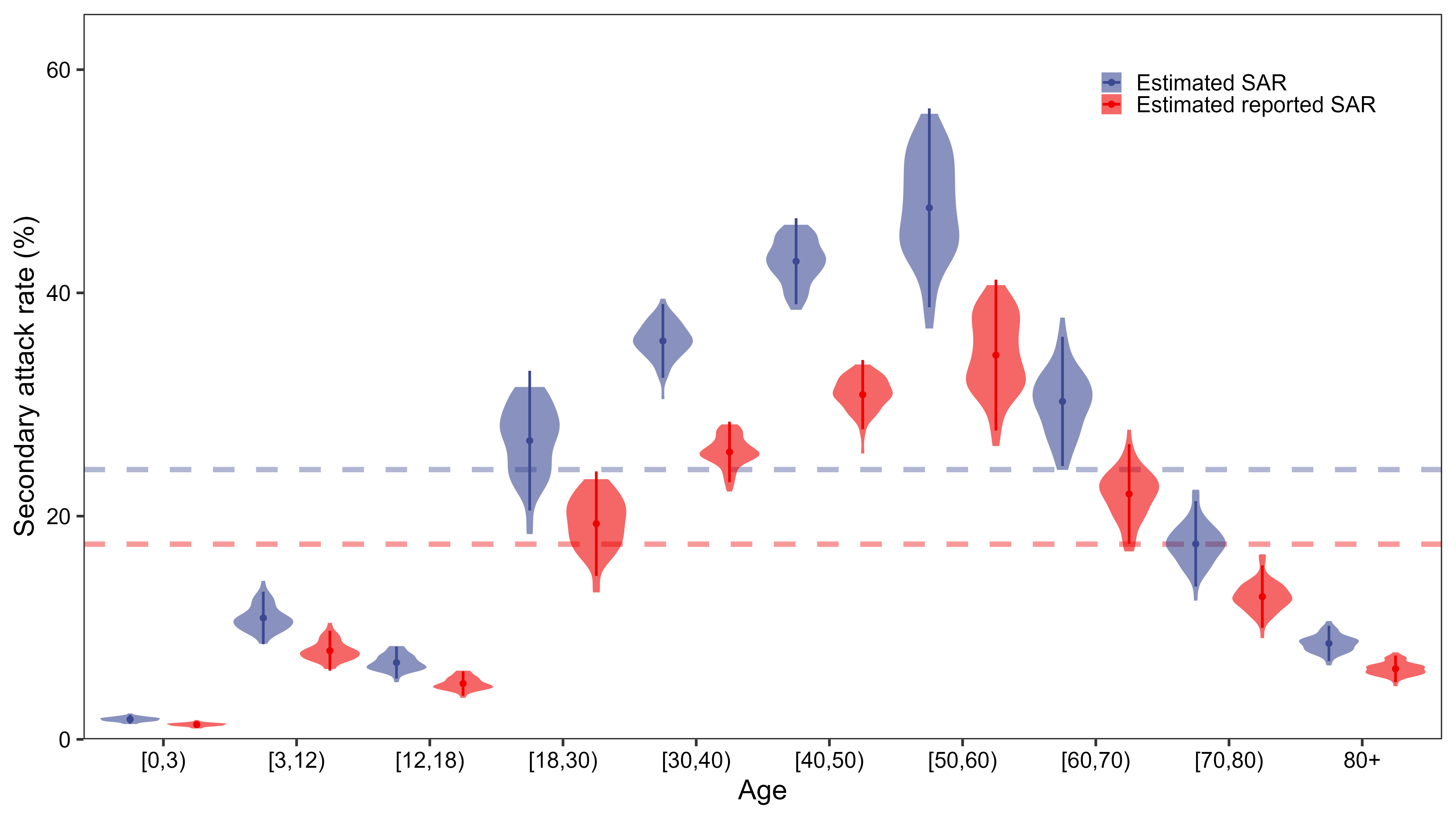
Figure S10. Comparation of estimated secondary attack rates (SAR) and estimated reported SAR (SAR_obs_). Blue and red dashed line is overall SAR (24.1%) and overall SAR_obs_ (17.4%).** The number of total contacts is same in the calculation of SAR and SAR_obs_, while the numerator was different. In calculation of SAR, the numerator is the all infections, including ascertained and unascertained cases. The numerator in calculation of SAR_obs_ is the ascertained cases (namely, the reported cases).
